# Chick chorioallantoic membrane (CAM) assay for the evaluation of the antitumor and antimetastatic activity of platinum-based drugs in association with the impact on the amino acid metabolism

**DOI:** 10.1016/j.mtbio.2023.100570

**Published:** 2023-01-31

**Authors:** Katerina Mitrevska, Miguel Angel Merlos Rodrigo, Natalia Cernei, Hana Michalkova, Zbynek Splichal, David Hynek, Ondrej Zitka, Zbynek Heger, Pavel Kopel, Vojtech Adam, Vedran Milosavljevic

**Affiliations:** a Department of Chemistry and Biochemistry, Mendel University in Brno, Zemedelska 1, CZ-613 00, Brno, Czech Republic; b Central European Institute of Technology, Brno University of Technology, Purkynova 123, CZ-612 00, Brno, Czech Republic; c Department of Inorganic Chemistry, Faculty of Science, Palacky University, 17. Listopadu 12, CZ-779 00, Olomouc, Czech Republic

**Keywords:** Platinum nanoparticles, Cisplatin, CAM assay, Breast cancer, Amino acids metabolism, TCA cycle

## Abstract

The combination of *in ovo* and *ex ovo* chorioallantoic membrane (CAM) assay provides an excellent platform which extends its relevance in studying carcinogenesis to the field of screening of anticancer activity of platinum nanoparticles (PtNPs) and further study of the amino acids’ fluctuations in liver and brain. PtNPs are promising candidates for replacing cisplatin (CDDP); however, insufficient data of their antitumor efficiency and activity on the cancer-related amino acid metabolism are available, and the assessment of the *in vivo* performance has barely scratched the surface. Herein, we used CAM assay as *in vivo* model for screening of novel therapeutic modalities, and we conducted a comparative study of the effects of CDDP and polyvinylpyrrolidone coated PtNPs on MDA-MB-231 breast cancer xenograft. PtNPs showed a higher efficiency to inhibit the tumor growth and metastasis compared to CDDP. The amino acids profiling in the MDA-MB-231 ​cells revealed that the PtNPs had an overall depleting effect on the amino acids content. Noteworthy, more side effects to amino acid metabolism were deduced from the depletion of the amino acids in tumor, brain, and liver upon CDDP treatment. Different sets of enzymes of the tricarboxylic acid (TCA) cycle were targeted by PtNPs and CDDP, and while mRNA encoding multiple enzymes was downregulated by PtNPs, the treatment with CDDP affected only two TCA enzymes, indicating a different mechanism of action. Taken together, CAM assay represents and invaluable model, demonstrating the PtNPs capability of repressing angiogenesis, decrease amino acid contents and disrupt the TCA cycle.

## Graphical abstract

**Figure:**
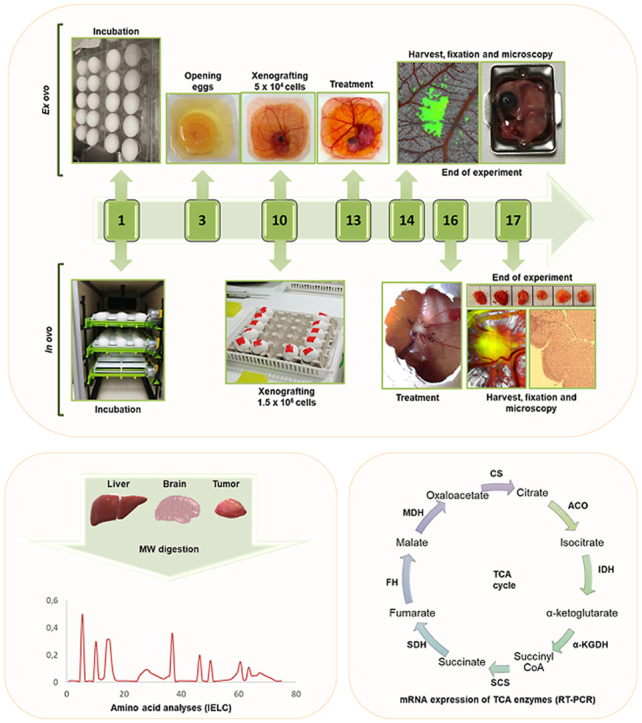

## 1 Introduction

In the past few decades, the platinum drugs have been the top contenders as chemotherapeutic agents in the treatment of various forms of malignant cancers. Cisplatin (CDDP) is one of the commonly used platinum derivatives, however, limitations to the use of CDDP are unfortunately present and numerous, from induction of dose-dependent side toxicity and drug resistance, to causing disfunction of many systems, as a result of developed nephrotoxicity, neurotoxicity, ototoxicity, hemolysis, and toxicity to the reproductive system [1]. Taking this into account, the search for alternative treatments has become an imperative action.

Nanotechnology has offered a great potential for developing functional nanomaterials with the potential to replace the conventional platinum drugs. In fact, the foundation of nanotechnology lays in manufacturing nano-sized materials such as nanoparticles (NPs), which differs in functionality compared to their bulk equivalents [2]. The implementation of noble metal NPs in medicine and as pharmaceuticals has been on the rise and progressed towards new means and resources for cancer treatments [3]. Platinum nanoparticles (PtNPs), the concurrent potential substitutes for CDDP, are still under investigation for their anticancer efficiency [4]. In general, the platinum-based drugs manifest their effect through DNA damage, by selectively binding Pt^2+^ ions to the purines of the DNA, resulting in breakage of the DNA helix and cease the replication and transcription [5]. While CDDP tends to covalently bind the DNA bases, hindering the DNA replication and transcription, the effect of PtNPs differs and mostly results from making complexes with DNA polymerase leading to its inhibition [5,6]. Recent studies have examined the *in vitro* cytotoxic effect in cancer cells cultures, and revealed that the PtNPs cause morphological and apoptotic changes as well as cell cycle arrest in breast cancer [1,3,7], human lung adenocarcinoma [8,9], ovarian teratocarcinoma [9], pancreatic cancer [9], hepatocellular carcinoma [10], and glioblastoma multiforme [11]. However, evidence of the antitumor effects of PtNPs obtained from *in vivo* investigations are insufficient, and the examinations are limited to the main antitumor effects against few types of cancers, without any insights in the side-effects to the healthy tissues.

One of the main features of the cancer cells that differentiate them against normal cells is the increased energy metabolism focused on production of electron transport reducing equivalents and ATP, especially in the tricarboxylic acid cycle (TCA) [12]. In its essence, the TCA cycle uses carbohydrates, fats, and amino acids as metabolic sources to produce energy, through the catalytic activity of the TCA enzymes whose expression is modulated in cancer cells to maximize the yields of the mitochondrial respiration [[13], [14], [15], [16]]. The amino acids are indispensable source fueling the TCA cycle, highlighting the importance of glutamine in cancer cells metabolism [17,18]. Besides supplying the TCA cycle, these protein building blocks are also involved in numerous signaling pathways, purine biosynthesis, lipogenesis, and they are involved in the regulation of the progressive induction of reactive oxygen species (ROS) in cancer cells, as sources for glutathione synthesis [[19], [20], [21], [22]]. Thus, it is critical to investigate the impact of the administered platinum drugs on the TCA cycle and the amino acids contents of the cancer cells and the tissues where the cancer cells have metastasized.

The chick chorioallantoic membrane (CAM) assay has attracted a lot of attention as a replacement for the murine model to evaluate the activity of novel drugs and bioactive molecules [23]. The benefits of the CAM assay go beyond simplicity and cost effectiveness, allowing efficient tumor cell xenografting and tumorigenesis during a short amount of time, observation of metastasis and angiogenesis, and evaluation of drug characterization and delivery [[24], [25], [26], [27], [28]]. Moreover, a well-established *in ovo* and *ex ovo* modifications are available, complementing each other, which provides a wide array of subsequent experimentations [29]. The CAM assay has been successfully applied for the study of various drugs and nanotoxicological implications, however, the study of PtNPs using this model has been restricted to the data regarding the effects of PtNPs on glioblastoma multiforme [11].

In our previous studies, PtNPs have shown a promising efficiency against MDA-MB-231 ​cell line, and the extent of the efficiency was related to the size of the PtNPs coated with polyvinylpyrrolidone (PVP) of different molecular weights, that is PtNPs-10 coated with PVP with molecular weight of 10,000 and PtNPs-40 coated with PVP with molecular weight of 40,000 [30]. Herein, we evaluated the antitumor efficiency of PtNPs-10, PtNPs-40 and CDDP against MDA-MB-231 primary tumors in chicken embryos employing *in ovo* and *ex ovo* CAM assay, in which we investigated anticancer effects as well as inhibitory activity toward angiogenesis and metastasis. Furthermore, we highlighted the impact of the treatment on the amino acid's contents in the MDA-MB-231 ​cell culture, primary tumors, and liver and brain, representing organs with confirmed metastatic colonization. Finally, as extension to the impaired amino acid metabolism, we identified the enzymes of the TCA cycle whose expression was deregulated by CDDP, PtNPs-10 and PtNPs-40.

## 2 Materials and methods

### 2.1 Chemicals

Listed chemicals were purchased from Sigma-Aldrich (St. Louis, MO, USA) in ACS purity, unless noted otherwise.

### 2.2 Synthesis and characterization of PVP-coated PtNPs

The PtNPs-10 and PtNPs-40 were synthesized by using protocol published in our previous work [30]. Briefly, 0.07 ​g of PtCl_4_ was dissolved in 10 ​mL water containing 33 ​μL of 37% HCl, whereupon 0.14 ​g of PVP with different molecular weight PVP-10k and PVP-40k was added for the synthesis of PtNPs-10 and PtNPs-40, respectively. Subsequently, 5 ​mL of H_2_[PtCl_6_] was added to the mixture stirring for 1 ​h. Finally, 50 ​mg of NaBH_4_ was added as a reduction agent and the filled up to 50 ​mL, with stirring for 2 ​h more. The PtNPs-10 and PtNPs-40 were subsequently characterized by Fourier-transform infrared spectroscopy (FTIR) recording the IR spectra on a FTIR Jasco FT/IR-4700 with ATR technique. Additionally, Transmission Electron Microscope (TEM) analyses were performed using the sample (≈4 ​μL) deposited onto 400-mesh copper grids coated with a continuous carbon layer. Dried grids were imaged by Tecnai F20 TEM (FEI, Eindhoven, Netherlands) at 120 ​kV.

### 2.3 Cell lines and culture conditions

A human breast cancer cell line established from a pleural effusion, MDA-MB-231, was used to study the effects of the selected platinum-based drugs. The cell line was purchased from the American Type Culture Collection (ATCC, Manassas, VA, USA). The cells were cultured in RPMI 1640 with 10% foetal bovine serum (FBS) and the media was supplemented with penicillin (100 U/mL) and streptomycin (0.1 ​mg/mL). The cells were maintained at 37 ​°C and 5% CO_2_ in a humidified incubator Galaxy® 170 ​R (Eppendorf, Hamburg, Germany).

### 2.4 Cell viability assay

MTT ((3-(4,5-dimethylthiazol-2-yl)-2,5-diphenyltetrazolium bromide)) assay was used to assess the susceptibility of MDA-MB-231 ​cells the newly synthesized PtNPs. MTT assay was applied by suspending 5,000 ​cells in 50 ​μL medium into each well of microtiter plates, with further incubation for 24 ​h at 37 ​°C with 5% CO_2_ to ensure cell growth. The effects on cell viability were determined for PtNPs-10, PtNPs-40 within concentration range of 0.01–25 ​μg/mL and the treatment was carried out for 24 ​h. Then, 10 ​μL of MTT [5 ​mg/mL in phosphate buffered saline (PBS)] was added to the cells and the mixture was incubated for 4 ​h at 37 ​°C. Next, the MTT-containing medium was replaced with 100 ​μL of 99.9% dimethyl sulfoxide (DMSO) and the absorbance of the samples after 5 ​min incubation was determined at 570 ​nm using Infinite 200 PRO (Tecan, Männedorf, Switzerland) [31].

### *2.5* *Ex ovo* chorioallantoic membrane assay

In this study, we followed the conditions of *ex ovo* CAM previously reported in our study [29]. The fertilized chicken eggs purchased from a local provider (INTEGRA, a. s., Zabcice, Czech Republic) were incubated with rotation at 37.5 ​°C and 65% humidity for 3 days. Before xenografting, MDA-MB-231 ​cells were pre-labeled with CellTracker Green (Invitrogen, Carlsbad, CA, USA), and implanted on the CAM at an initial seeding density of ∼5 ​× ​10^4^. After of incubation of 3 days, 5 ​μL of 100 ​μg/mL CDDP or 250 ​μg/mL PtNPs-10 or PtNPs-40 was added to each microtumor and *ex ovo* cultures were further incubated at 37.5 ​°C for 24 ​h. For fluorescent angiography, 50 ​μL of 5 ​μg/mL of rhodamine-labeled *Lens culinaris agglutinin* (LCA) (Vector laboratories, Burlingame, CA, USA) was injected in the peripheral veins of the viable CAM using a 30G hypodermic needle attached to a 1 ​mL syringe. After the injection, the embryos were incubated for another 3 ​min to let the LCA circulate in the bloodstream and then, the embryos were sacrificed by cutting the vitelline arteries. CAM areas with microtumors were cut with a 3 ​cm margin around them and fixed in 3.7% paraformaldehyde (Sigma Aldrich, St. Louis, MO, USA) in PBS, and the embryos were fixed in the same manner. For subsequent fluorescent angiography, the EVOS FL Auto Cell Imaging System (Thermo Fisher Scientific) was used with the emitted light from rhodamine collected in a detection window 580 ​nm by Texas Red light cube (Thermo Fisher Scientific, Waltham, MA, USA), and green light from MDA-MB-231 ​cells labeled with CellTracker Green collected at 488 ​nm by GFP light cube (Thermo Fisher Scientific). The relative area (%) of the tumors was quantified by ImageJ software. To confirm the development of a 3D tumor we prepared cross-section images of the untreated tumors by EVOS and we carried out confocal laser scanning microscopy (CLSM) (LSM 880, Carl Zeiss, Jena, Germany) in 3D mode. Using fluorescent macroscopy, MDA-MB-231 ​cells were visualized into the chick embryos using the Azure 600c (Azure Biosystems, Dublin, CA, USA) equipped with the three RGB fluorescence channels for applications detecting fluorescent biomolecules in the visible range, Cy2/Cy3/Cy5 [32]. In the EU countries, CAM assay is not declared as an animal experiment by law, and therefore, does not require ethical approval.

### *2.6* *In ovo* chorioallantoic membrane assay

To study the efficiency of PtNPs-10 and PtNPs-40 to inhibit primary MDA-MB-231 tumor growth and metastasis in the organs, we followed the protocol described in our previous study [29]. 25 ​μL of the cell suspension containing ∼1.5 ​× ​10^6^ MDA-MB-231 ​cells were grafted near the allantoic vein bifurcation without touching the CAM. Before xenografting, the MDA-MB-231 ​cells were pre-labeled with CellTracker Green (Invitrogen, Carlsbad, CA, USA) and the eggs were incubated for 6 days at 37.5 ​°C. Then, 25 ​μL of 100 ​μg/mL CDDP or 250 ​μg/mL PtNPs-10 or PtNPs-40 was added topically on the upper CAM and the eggs were incubated for additional 24 ​h. At indicated time-points, 50 ​μL of 10 ​μg/mL of LCA (Vector laboratories, Burlingame, CA, USA) was injected in the peripheral veins of the viable CAM using a 30G hypodermic needle attached to a 1 ​mL syringe, to fluorescently label the blood circulation. Then, portions of the CAM distal, liver and brain were harvested to perform additional analyses, and to locate the human tumor cells which colonized the tissues. EVOS FL Auto Cell Imaging System (Thermo Fisher Scientific, Waltham, MA, USA) was used to detect the emitted green light from MDA-MB-231 ​cells labeled with CellTracker Green, collected at 488 ​nm and from rhodamine collected in a detection window 585 ​nm in distal CAM. To locate the metastasizing human tumor cells in chick brain and liver by EVOS FL Auto Cell Imaging System (Thermo Fisher Scientific, Waltham, MA, USA), we observed the cells in glass slides containing manually pressed tissue samples, adapted from the protocol published by Augustine *et al*., [33]. For the purposes of observing the all tissue as well, nuclei were counterstained with Hoechst 33 ​258.

### 2.7 Histopathology

Paraffin blocks were produced for each *in ovo* CAM tumor, and *ex ovo* embryos. The histological sections were first deparaffinized and hydrated in xylene and graded alcohol series and subsequently stained with hematoxylin and eosin (H & E) according to standard protocol. Histological images were acquired by using EVOS FL Auto Cell Imaging System (Thermo Fisher Scientific).

### 2.8 Ion-exchange liquid chromatography (IELC) analyses for the amino acids quantification in MDA-MB-231 ​cell suspension, primary tumors, liver and brain

The samples treated with CDDP, PtNPs-10, PtNPs-40 and without treatment (Control) were processed by acidic hydrolysis of 25 ​mg solid tissue or MDA-MB-231 ​cell suspension. Briefly, 0.5 ​mL of 6 ​M HCl was added to the samples, and they were subsequently digested in a microwave reactor Anton Paar (Anton Paar GmbH, Graz, Austria) under controlling conditions for 90 ​min (power 80 ​W, temperature 120 ​°C and maximum pressure of 25 ​bar). The samples were then centrifuged (Centrifuge Z326 K, Hermile, Germany) at 4 ​°C, 24,000 ​g, 10 ​min, and 100 ​μL of hydrolysed sample was diluted with 900 ​μL of dilution buffer (5 ​mL/L of thiodiglycol, 14 ​g/L of citric acid, 11.5 ​g/L of sodium chloride) and centrifuged at 4 ​°C, 24,000 ​g, 10 ​min. The samples were then diluted with 500 ​μL of 0.6 ​M NaOH in the dilution buffer and used for further amino acid analysis.

The determination of amino acids content in cells and tissues before and after application of CDDP, PtNPs-10 and PtNPs-40 was conducted by IELC (Model AAA-400, Ingos, Prague, Czech Republic) equipped with UV/Vis light absorption detector and post column derivatization by ninhydrin. The glass column had an inner diameter of 3.7 ​mm and 350 ​mm length, and it was filled with strong cation exchanger with average size of particles around 12 ​μm with 8% porosity. The double channel UV/Vis light absorption detector was set to 440 and 570 ​nm and 60 ​°C was set as the working temperature of the column. The solution of ninhydrin in 75% v/v methylcelosolve (Ingos, Prague, Czech Republic) and 2% v/v 4 ​M acetic buffer (pH 5.5) was used as post column derivatization agent, whereas SnCl_2_ was used as a reducing agent. The buffer used for the elution of the amino acids was composed of 10.0 ​g of citric acid, 5.6 ​g of sodium citrate, and 8.36 ​g of NaCl per liter of solution and pH was 3.0 and flow rate of 0.25 mL/min was applied.

### 2.9 Isolation of RNA

MDA-MB-231 ​cells were seeded into 12-well plates (2 ​× ​10^5^/well) and allowed to settle and grow overnight. Subsequently, subconfluent cells were treated (except the control) with CDDP (10 ​μg/mL) or PtNPs (25 ​μg/mL) for 24 ​h. Afterward, cells were harvested for RNA isolation (mixture of cells from 3 wells ​= ​one replicate). The experiment was repeated three times. RNA isolation was performed by the RNeasy Mini Kit purchased from QIAGEN (Hilde, Germany) according to the manufacturer's instructions. All samples were treated by DNase from RNase-Free DNase Set (QIAGEN, Hilde, Germany) and eluted in 40 ​μL of RNAse-free water. The concentration and purity of isolated RNA were measured spectrophotometrically by NanoDrop™ One/OneC Microvolume UV/Vis Spectrophotometer (Thermo Fischer Scientific, Waltham, USA). RNA integrity was verified using a bleach gel [34]. 1 ​μg of each RNA sample were separated for 90 ​V/25min in 1% agarose gel stained by ethidium bromide (0.5 ​μg/mL) supplemented with 1% bleach. The gel was visualized by Azure c600 from Azure Biosystems (Dublin, California, USA) (Fig. S1).

### 2.10 RT-qPCR

Synthesis of cDNA was achieved with the First Strand cDNA Synthesis Kit from Roche (Basel, Switzerland) according to the manufacturer's instructions. 1,000 ​ng of total RNA was transcribed using random hexamer primers. After reverse transcription, cDNA (20 ​μL) was diluted in 780 ​μL of UltraPure™ DNase/RNase-Free Distilled Water (Thermo Fisher Scientific, Waltham, USA) to a final concentration of 1.25 ​ng/μL. For qPCR was used 5 ​μL of diluted cDNA/reaction.

Reference sequences (RefSeq) from NCBI's database (http://www.ncbi.nlm.nih.gov/RefSeq/) were used as the template for primer design. Target-specific primers (Table S1) flanking the intron or spaning the exon-exon junctions (except *BCAT1* primer set) were designed by PrimerQuest™ Tool (Integrated DNA Technologies, Coralville, USA). The amplicon size and control of primer-dimer formation were checked by gel electrophoresis (2.5% agarose gel stained by EthBr, 90 ​V, 60 ​min) and visualized by Azure c600 from Azure Biosystems (Dublin, California, USA) (Fig. S2).

Analysis of relative gene expression was performed by real-time PCR (qPCR) method using qTOWER³ Touch from Analytik Jena (Jena, Germany). For each reaction (10 ​μL), 5 ​μL of diluted cDNA and 4.5 ​μL of Luna® Universal qPCR Master Mix (New England Biolabs, Ipswich, Massachusetts, USA) with 250 ​nM (0.25 ​μL of 10 ​μM stock solution) of forward and reverse primer was mixed. The qPCR program was performed as follows: initial denaturation at 95 ​°C for 5 ​min and subsequent 40 cycles of denaturation at 95 ​°C for 20 ​s and extension at 60 ​°C for 30 ​s. The qPCR reaction was followed by melting curve analysis to check the Tm of qPCR products and validate the amplification specificity (Fig. S3).

The threshold cycle (CT) was determined by qPCRsoft 4.0 from Analytik Jena (Jena, Germany). *GAPDH* (glyceraldehyde-3-phosphate dehydrogenase), *PGK1* (phosphoglycerate kinase 1), and *RPLP0* (ribosomal protein lateral stalk subunit P0) were tested as reference genes. To evaluate the stability of all selected reference genes was used BestKeeper algorithm (Fig. S4; Table S2) [35]. As the most stable reference gene was selected *RPLP0* which was then used for the normalization of gene expression (ΔCT ​= ​CT_*RPLP0*_ - CT_GOI_).

### 2.11 Statistical analysis

Statistical analysis was performed by Student's t-test and one-way ANOVA followed by Dunnett's post hoc or Tuckey's post hoc test using GraphPad Prism version 8.0.1 (GraphPad Software, CA, USA). A p-value less than 0.05 (typically ≤0.05) was considered statistically significant.

## 3 Results

### 3.1 Characterization of PtNPs-10 and PtNPs-40

The size and the morphology of the PtNPs-10 and PtNPs-40 were determined by TEM. A spherical shape of the synthetized PtNPs was predominant and the individual particle size was approximately 10 ​± ​2 ​nm (Fig. S5), demonstrating a successful synthesis of the nanoparticles with uniform size and good dispersion. The FTIR spectroscopy results revealed the nature of the interactions between PVP and the surface PtNPs (Fig. S6). The presence of identical band at 1573 ​cm^−1^ attributed to the carbonyl stretch (C=O) are present only in case of PtNPs-10 and PtNPs-40, while the same band is not present in pure PVP. This suggested that a contact between pyrrolidone rings from PVP and the platinum surface occurred. Numerous studies suggest that PVP interacts with Pt via oxygen–metal bond formation [36,37]. Ye *et al*., reported that the direction of the charge transfer goes from the carbonyl group to platinum which is dependent on the size of particles. In case of larger particles (≥25 ​nm) the charge is going to opposite direction [36]. We obtained similar results in our previous published work, where the binding energy of C=O group was associated with the electron emission from the carbon atom of the amide group carbonyl substituent, indicating the direct bonding of carbonyl to the platinum surface and charge transfer from carbonyl group to platinum [38].

### 3.2 Assessment of the effect of CDDP, PtNPs-10 and PtNPs-40 by *ex ovo* chorioallantoic membrane assay

**Fig. 1:**
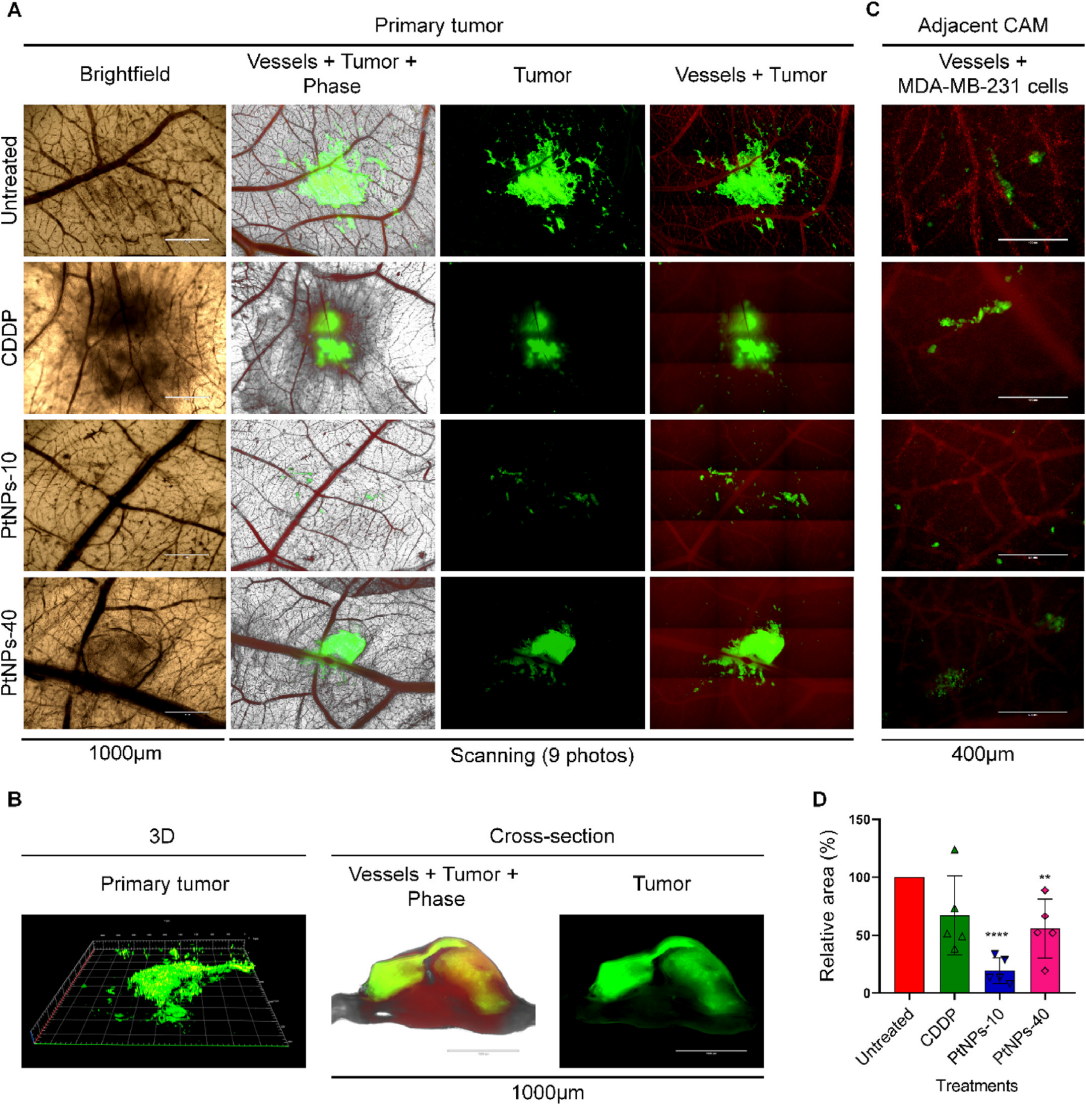
*Ex ovo-*cultivated chicken embryos. (A) Fluorescent microscopic imaging of the tumor on the area of implantation of the MDA-MB-231 ​cells on the chick chorioallantoic membrane (CAM) treated with CDDP, PtNPs-10, PtNPs-40, and untreated tumors during 24 ​h. Viable MDA-MB-231 ​cells are green (labeled with CellTracker Green) and angiogenic vessels are red (labeled with rhodamine *Lens culinaris* agglutinin (LCA). Scale bars represent 1000 ​μm. Photos without scale bars represent scanning (3 ​× ​3, 1,000 ​μm per each photo). (B) 3D micrograph and a cross-section (transversal view) of the untreated microtumor by fluorescent microscopy. (C) Invasive vasculotropic MDA-MB-231 ​cells that escaped from primary tumor site and extravasated in the adjacent CAM. Scale bars represent 400 ​μm. (D) Quantification of the relative area (%) of the microtumors by ImageJ software (∗∗p ​< ​0.005, ∗∗∗∗p ​< ​0.0001).

We first validated the susceptibility of MDA-MB-231 ​cells to PtNPs. MTT assay was conducted applying a 24 ​h treatment with PtNPs in a concentration range of 0.1–25.0 ​μg/mL, and concentration of 25.0 ​μg/mL was recorded as a concentration responsible for the reduction of half of the cell population, whereas for the commercial CDDP we used previously established concentrations on MDA-MB-231 (Fig. S7) [30]. The *ex ovo* CAM assay was conducted to study the inhibitory effects of CDDP and PtNPs on the growth of MDA-MB-231 xenograft in *ex ovo* CAM assay. Fig. 1A demonstrates that the MDA-MB-231 xenografts were properly established on the upper CAM, which was observed as extensive green signal of the MDA-MD-231 ​cells labeled with CellTracker. However, the tumors have an appearance of a monolayer, rather than a 3D tumor, since the samples are observed from the bottom side of the CAM, while the tumor is on opposite side on the surface of the CAM. To validate the formation of a 3D primary tumor, we prepared cross-section images of the untreated tumors, and we scanned the area of interest by CLSM in 3D mode, thus we detected the fluorescent signal extending in three dimensions (Fig. 1B). This demonstrated that the MDA-MB-231 ​cells are not simply arranged as a monolayer on the surface of the CAM, but an actual 3D tumor has been developed. Moreover, the vasculature of the embryo, as well as cancer cells can be simultaneously observed to evaluate the formation of metastasis including the extravasation of the cells towards the adjacent CAM (Fig. 1C). In addition, the relative area of the MDA-MB-231 tumors was quantified by ImageJ software showing that the MDA-MB-231 tumors which were treated with PtNPs and CDDP displayed a distinct reduction of the primary tumor size (Fig. 1D). Upon 24 ​h of exposure to PtNP-10, the MDA-MB-231 tumors were disintegrated, and the adjacent CAM presented less evidence of cancer cells. The obtained results confirmed the chemo-sensibility of MDA-MB-231 ​cells to PtNP-10 and the reliability of *ex ovo* CAM assay for *in vivo* screening of PtNPs with anticancer activity.

**Fig. 2:**
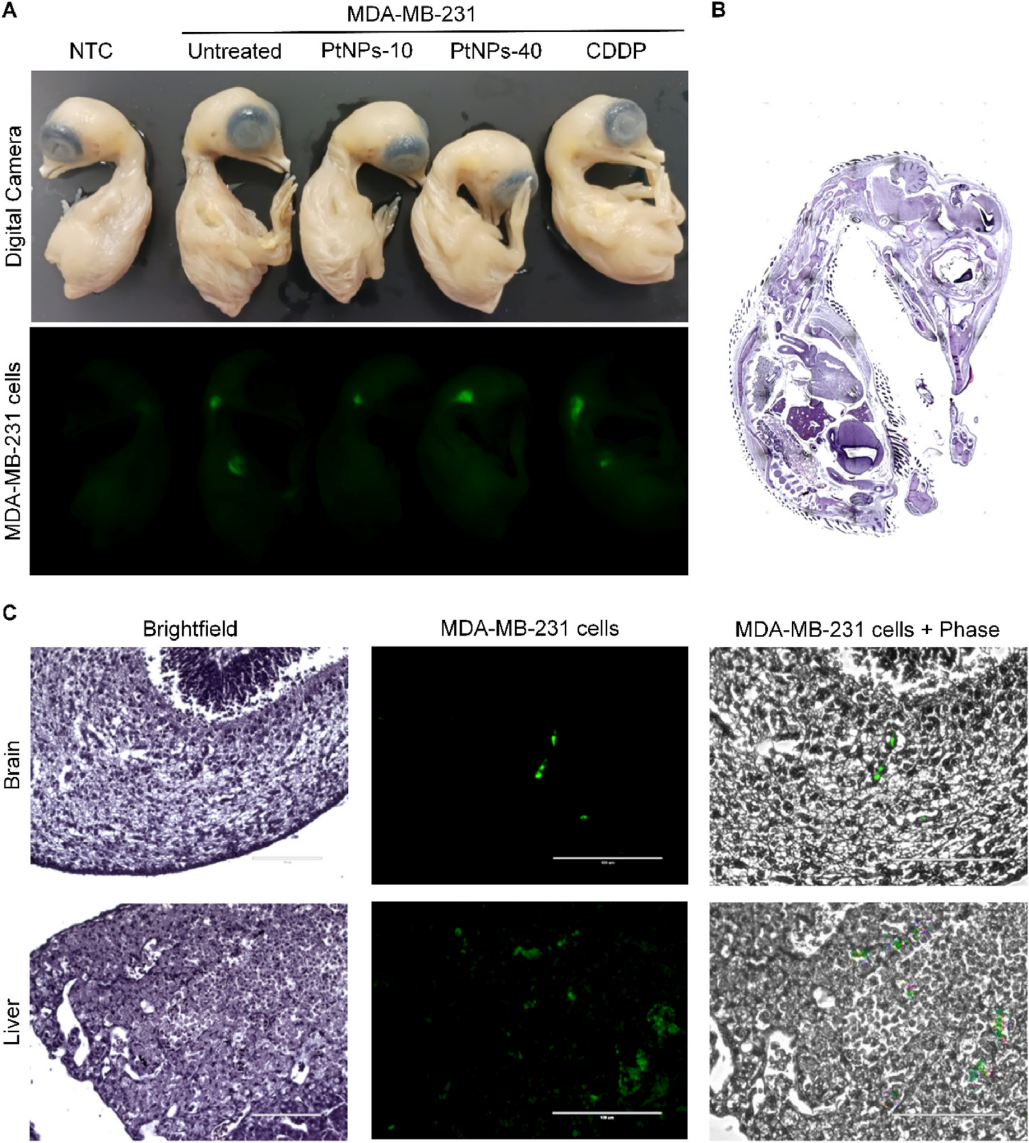
Fluorescent visualization of MDA-MB-231 migration and colonization in the chick embryos by *ex ovo* CAM assay. (A) Green fluorescent macroimaging of the MDA-MB-231 ​cells by Azure 600c. NTC, embryo without MDA-MB-231 xenograft. (B) H & E staining of paraffin sections of whole embryo 4 days post MDA-MB-231 ​cells xenografting. Photo without scale bars represent scanning (98 photos, 1,000 ​μm per each photo). (C) Tissue section showing green fluorescence of MDA-MB-231 ​cells in brain and liver tissue (scale bar 1,000 ​μm).

The following analysis involved visualization of the migration of the MDA-MB-231 ​cells from primary tumors throughout the whole embryo, and their extravasation and colonization of other tissues (Fig. 2). The sacrificed *ex ovo* embryos were fixed and macroscopic visualization of the whole embryo was achieved by Azure 600c to detect fluorescently labeled MDA-MB-231 (Fig. 2A). However, the whole embryo presents a high level of autofluorescence by itself, which hampered with the proper distinction of the metastasized cells. To overcome this issue, we arranged the embryos by placing the embryo of the negative control (not xenografted with MDA-MB-231) and the xenografted embryos side by side, and we adjusted the autofluorescence of the negative control, reducing it to non-existent levels. Thus, the autofluorescence of the xenografted embryos was reduced as well, allowing to observe only the fluorescent signals of the MDA-MB-231 ​cells. The overall effect of the treatments is presented in Fig. 2A where it indicates that the highest potential for inhibition of metastasis has been delivered by PtNPs-10, followed by PtNPs-40. In contrast, the treatment with CDDP failed to reduce metastatic colonization, or only minor effects were achieved. Similar methodology was carried out by Pawlikowska *et al*. [32]. To further validate the green fluorescence detected by Azure, we prepared H & E staining of paraffin sections of the *ex ovo* embryos (Fig. 2B), and the exact tissues where the metastatic colonization was most prominent, were identified by the fluorescent signals, indicating that MDA-MB-231 ​cells possess markedly higher ability to spread to liver, and brain (Fig. 2C). To induce more substantial metastatic migration and colonization to ensure stronger fluorescence, we extended the *ex ovo* assay into *in ovo* assay where a higher initial concentration of the MDA-MB-231 ​cells was applied, which promoted a growth of a macroscopically visible tumor, and consequently enhanced the development of metastasis in other organs.

### 3.3 Assessment of the effect of CDDP, PtNPs-10 and PtNPs-40 by *in ovo* chorioallantoic membrane assay

The *ex ovo* set up was carried out with the purpose of creating a microtumor by introducing a concentration of 5 ​× ​10^4^ MDA-MB-231 ​cells, which subsequently lead to migration of the MDA-MB-231 ​cells from the microtumor on the CAM to more distant locations inside the embryo and extravasation into other organs. In the next step, we used *in ovo* CAM assay to investigate the ability of CDDP, PtNPs-10 and PtNPs-40 to suppress the growth of MDA-MB-231 primary tumor, intravasation, extravasation and metastatic spreading of MDA-MB-231 ​cells *in vivo*. Unlike the *ex ovo* instalment, the *in ovo* CAM assay was carried out by applying a higher initial concentration of 1.5 ​× ​10^6^ of the MDA-MB-231. By doing so, we promoted a growth of a macroscopically visible tumor, and consequently enhanced the intravasation and migration in the chick's circulatory system to more distant locations from the primary tumor (distal CAM) and extravasation into other organs. This method provided us with a convenient means for collection of the tumors and distal CAM, which could be analyzed on a microscopic and macroscopic scale, as well as brain, and liver which were subjected to further analyses. The larger-scale tumors induced by applying higher initial concentration of MDA-MB-231 ​cells, reinforced a larger fluorescent signal of the tumor itself, as well as an enhanced metastatic colonization in other organs which provided an intensified fluorescence for a more efficient detection of the metastasis.

**Fig. 3:**
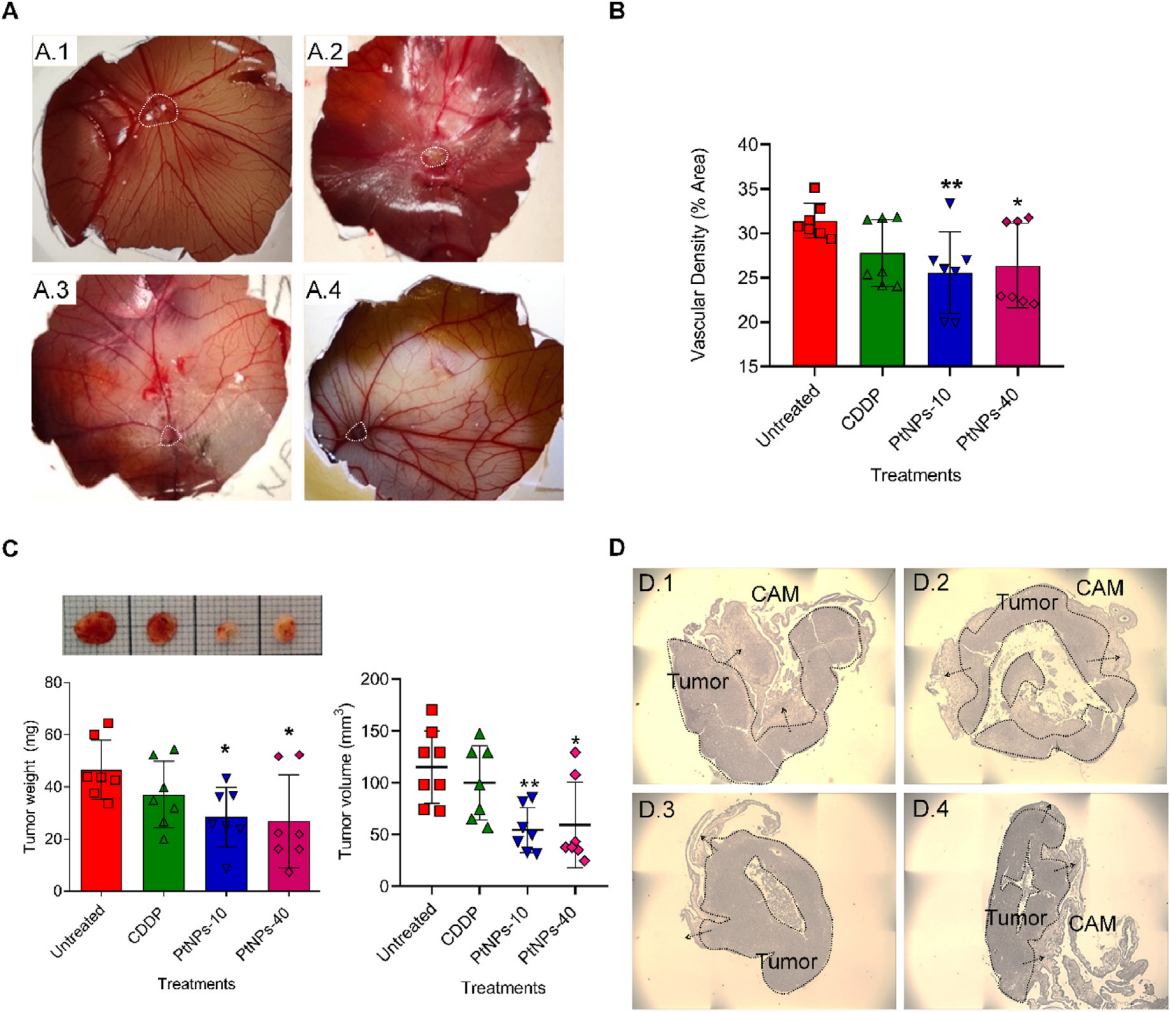
*In ovo* CAM assay. (A) Photographs of the CAM (upon experiment termination on the 17^th^ day); Untreated (A.1), CDDP (A.2), PtNPs-10 (A.3), and PtNPs-40 (A.4). Microtumors are demarcated by white dashed lines. (B) Vascular density (% area) calculated by Vessel Analysis ImageJ software (∗p ​< ​0.05, ∗∗p ​< ​0.005). (C) Photo of the tumors, tumor weights (mg) and tumor volume (mm^3^) after excision from the CAM (upon experiment termination on the 17^th^ day). Data show mean ​± ​SEM (n ​= ​7); ∗p ​< ​0.05, ∗∗p ​< ​0.005. (D) H & E staining of spontaneous MDA-MB-231 tumors. The arrows indicate invasion zones in the CAM; Untreated (D.1), CDDP (D.2), PtNPs-10 (D.3), and PtNPs-40 (D.4). Photos without scale bars represent scanning. (3 ​× ​3, 1000 ​μm each photo).

To elaborate in detail, we induced MDA-MB-231 xenografts in *in ovo* CAM for 6 days as shown in Fig. 3A. Then, we treated the *in ovo* MDA-MB-231 tumors with CDDP and PtNPs during 24 ​h. By applying this method, we were able to directly observe the tumor on the surface of the CAM, where the blood vessels were strongly oriented and concentrated around the tumor (Fig. 3A). To assess the extent of the vessels’ network and the effects of the PtNPs treatments on the inhibition of angiogenesis, we calculated the vascular density (% area) by vessel analysis ImageJ software (Fig. 3B). The PtNPs-10 treatment was the most successful in diminishing the vascular density, followed by PtNPs-40, both of which managed to induce significant changes.

Upon termination, the assessment of the MDA-MB-231 tumor growth was realized through weight and volume measurements, demonstrating significant inhibitory activity of PtNPs-10 (approximately two-fold decline of the primary tumor weight and volume) and PtNPs-40 (approximately 1.5-fold decline of the primary tumor weight and volume) after 24 ​h (Fig. 3C) compared with the untreated samples. The results were consistent with the effects to the vascular density, demonstrating highest efficiency in tumor growth inhibition by PtNPs-10, whereas the efficiency of tumor inhibition by CDDP was limited (non-significant). The H & E staining of MDA-MB-231 xenografts excised from *in ovo* CAM assay validated the correct formation of tumors on the CAM (Fig. 3D). Furthermore, the H & E staining of the tumor sections showed that the cells migrate from the primary tumor, invading the nearby CAM. The effects of the CDDP and PtNPs treatments was visible as a partial disintegration of the primary tumor (Fig. 3D).

**Fig. 4:**
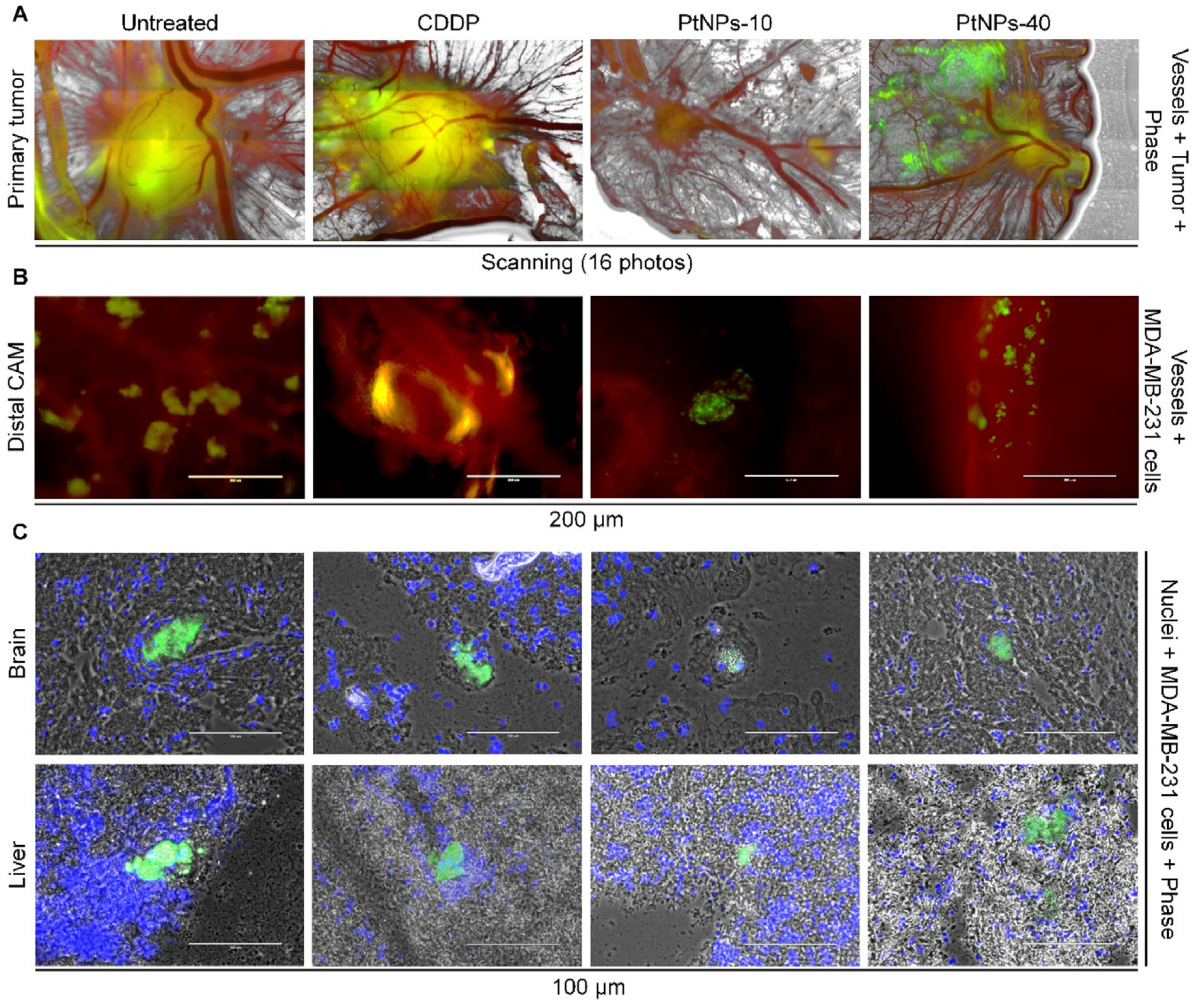
Fluorescent microscopic imaging of the primary tumor in the CAM (A) (upon experiment termination on the 17^th^ day), distal CAM (B) and brain and liver (C) after treatments with CDDP, PtNPs-10 and PtNPs-40 during 24 ​h. Viable MDA-MB-231 ​cells are green (labeled with Green CellTracker), angiogenic vessels are red (labeled with rhodamine *Lens culinaris* agglutinin (LCA)) and nuclei are blue (Hoechst 33 ​258 nuclei counterstain)). Photos without scale bars represent scanning (4 ​× ​4, 1,000 ​μm each photo).

Prior to the xenografting, the MDA-MB-231 ​cells were pre-labeled with CellTracker Green, which we used to visualize the tumor and its expansion into the surrounding CAM, by detecting the fluorescent signal of the MDA-MB-231 ​cells. For this purpose, the tumors were initially harvested together with a larger portion of the adjacent CAM, and as such, they were subjected to fluorescent microscopy. Fig. 4A Portrays comparable effects to the previously established inhibitory action of the PtNPs treatments. The size of the tumors after 24 ​h treatment with CDDP did not mark a noticeable reduction, and the blood vessels supplying nutrients to the tumor were kept almost intact. PtNPs-40 induced a visible degradation of the tumor into smaller fragments scattered around the primary tumor. However, in the case of PtNPs-10, a substantial shrinkage of the tumor was evident. Another valuable feature of *in ovo* CAM is that it allows a simple detection of intravasated and extravasated cells through fluorescent signals in the distal CAM, liver, and brain tissue (Fig. 4B and C). As shown in Fig. 4B, CDDP and PtNPs, displayed significant detection of extravasation and intravasation of the cells in CAM distal. In addition, PtNPs-10 exhibited higher inhibitory activity against migration to the distal CAM (Fig. 4B). To confirm the metastatic colonization from the MDA-MB-231 ​cells stained previously by Green CellTracker in chick brain and liver, which was initially observed in the *ex ovo* experiment, we took small pieces of each organ and pressed them between microscopic glasses and then the fluorescent signals were observed. The MDA-MB-231 ​cells were found assembled in groups among the chick cells of brain and liver tissue, producing a strong green fluorescence from the CellTracker, and were surrounded by normal cells whose nuclei were observed due to the blue staining with Hoechst 33 ​258 (Fig. 4C). To eliminate the possibility of autofluorescence in the organs, we prepared microscopic glasses of the liver and brain of the embryos which were not xenografted with MDA-MB-231 ​cells (negative control), and the presence of the blue stained nuclei of the healthy cells was confirmed, however, a green fluorescence was not observed in the samples (Fig. S8). Using this approach, we could not determine the proper degree of the metastasis, since only small portions of the tissues were analyzed. However, we found out that the fluorescent signals of the MDA-MB-231 ​cells in the tissues upon a treatment with PtNPs-10 were slightly inferior that the other treatments and more sporadically found, whereas in the tissues of the embryos treated with PtNPs-40 and CDDP the fluorescent signals were more frequent. Nevertheless, for the purpose of additional examination of the metabolic effects of the PtNPs in organs where metastatic migration occurred, this confirmation of metastasis in such organs was sufficient to identify liver and brain as the organs of interest.

### 3.4 Determination of the effect of CDDP, PtNPs-10 and PtNPs-40 on the amino acid profile

The metabolism of cancer cells is known to exhibit different patterns than normal cells, to maximize the utilization of the necessary nutrients to ensure enhanced multiplication and growth. Since the amino acids are essential supporters of the metabolic machinery of cancer cells, we explored how the different treatments affect the amino acid contents of *in vitro* culture of MDA-MB-231 ​cells and *in vivo* primary tumors formed on the surface of the CAM of a chicken embryo. In addition to the cancer cells and primary tumors, we analyzed the brain and liver tissues with metastatic colonization to determine the effect of CDDP and PtNPs on the amino acid profiles and whether they cause any concerning side-effects.

#### 3.4.1 The effect of CDDP, PtNPs-10 and PtNPs-40 on the amino acid profile of *in vitro* MDA-MB-231 ​cell culture

The treatment of MDA-MB-231 ​cell cultures produced a contrasting result between CDDP, PtNPs-10 and PtNPs-40 treated samples, which were compared to the untreated cells (Table 1). The treatment with CDDP caused a general increase of all amino acids, except for Pro and Cys, and significant changes were the increases of the concentrations of Thr, Ser, Ala and His. In contrast, the amino acid profile of MDA-MB-231 ​cells upon the treatment with PtNPs-10 displayed a lower concentration of amino acids (except Pro, Cys and Met), and the following amino acids Asp, Thr, Glu, Gly, Ala, Val, Ile, and Leu showed a significantly decreased values compared to the control group. Regarding the treatment with PtNPs-40, a similar pattern as in PtNPs-10 treatment was observed, displaying a decrease of almost all amino acids, however, Leu was the only amino acid which noted any significant differences.

**Table 1:** Concentration of amino acids in the MDA-MB-231 ​cells. Data is shown as mean difference of treated and untreated samples in a 95% (CI) and standard error (SE); n ​= ​3.

Amino acid	Mean difference (μmol/L) at 95% CI			SE
	CDDP vs. Untreated	PtNPs-10 vs. Untreated	PtNPs-40 vs. Untreated	
Asp	39.15 (-9.62 to 87.92)	-118.00 (-166.77 to -69.23)∗∗∗	-40.81 (-89.58 to 7.96)	15.23
Thr	23.83 (8.99–38.68)∗∗	-22.29 (-37.14 to -7.44)∗∗	-6.71 (-21.56 to 8.14)	4.64
Ser	53.09 (39.79–66.41)∗∗∗	-2.98 (-16.29 to 10.34)	2.06 (-11.26 to 15.37)	4.16
Glu	69.10 (-23.94 to 162.14)	-163.26 (-265.30 to -70.22)∗∗	-64.92 (-157.96 to 28.12)	29.05
Pro	-1.19 (-34.96 to 32.57)	6.45 (-27.31 to 40.22)	4.51 (-29.25 to 38.27)	10.54
Gly	37.96 (-56.60 to 132.51)	-150.52 (-254.07 to -55.96)∗∗	-47.44 (-141.00 to 47.12)	29.53
Ala	43.60 (2.93–84.28)∗	-86.79 (-127.46 to -46.11)∗∗∗	-34.32 (-74.00 to 6.35)	12.70
Cys	-0.52 (-142.01 to 140.97)	64.14 (-77.35 to 205.63)	1.04 (-140.45 to 142.53)	44.18
Val	24.55 (-30.98 to 80.08)	-85.38 (-140.90 to -29.84)∗∗	-42.30 (-97.83 to 13.23)	17.34
Met	6.47 (-56.41 to 69.36)	20.91 (-41.97 to 83.80)	-1.80 (-64.68 to 61.09)	19.64
Ile	49.30 (-7.84 to 106.44)	-61.57 (-118.71 to -4.43)∗	-0.30 (-57.44 to 56.84)	17.84
Leu	20.76 (-13.61 to 55.13)	-98.49 (-132.86 to -64.12)∗∗∗	-41.30 (-75.67 to - 6.93)∗	10.73
Tyr	23.48 (-28.39 to 75.36)	-24.43 (-73.30 to 27.45)	-16.86 (-68.73 to 35.02)	16.20
Phe	24.43 (-11.87 to 60.73)	-36.03 (-72.33 to 0.27)	-32.06 (-68.26 to 4.25)	11.34
His	186.35 (24.27–348.43)∗	-37.52 (-199.60 to 124.56)	-71.25 (-233.32 to 90.83)	50.61
Lys	263.44 (-22.55 to 747.42)	-270.80 (-754.79 to 213.18)	-163.44 (-647.43 to 320.54)	151.13
Arg	133.40 (-548.58 to 815.37)	-569.07 (-1251.05 to 112.90)	-231.78 (-913.76 to 450.19)	212.96

Negative sign in front of the mean indicates a decrease in the concentration of the amino acid in the treated sample compared to the control.

∗p ​< ​0.05, ∗∗p ​< ​0.01, ∗∗∗p ​< ​0.001.

#### 3.4.2 The effect of CDDP, PtNPs-10 and PtNPs-40 on the amino acid profile of MDA-MB-231 primary tumor in chicken embryo

To evaluate the effect of CDDP and PtNPs on the amino acids’ profile *in vivo*, we resected the MDA-MB-231 tumors from the CAM and analyzed the amino acid profile using IELC. Contrary to the findings from the *in vitro* experiment, the treatment with CDDP lead to a significant decrease of most of the amino acids in the tumor (Table 2). The effects of the treatment with PtNPs-10 and PtNPs-40 showed a resembling pattern, that is an overall depletion of most of the amino acids, however significant decreases were observed only for Cys and His upon the treatment with PtNPs-10. The treatment with PtNPs-40 resulted in a significant decrease of Thr, Ser, and His and increase in the contents of Arg.

**Table 2:** Concentration of amino acids in the tumors. Data is shown as mean difference of treated and untreated samples in a 95% (CI) and standard error (SE); n ​= ​9.

Amino acid	Mean difference (g/kg) at 95% CI			SE
	CDDP vs. Untreated	PtNPs-10 vs. Untreated	PtNPs-40 vs. Untreated	
Asp	-2.84 (-4.59 to -1.09)∗∗∗	-0.22 (-1.97 to 1.52)	-0.48 (-2.23 to 1.27)	0.64
Thr	-1.03 (-1.44 to -0.62)∗∗∗	-0.29 (-0.70 to 0.11)	-0.50 (-0.91 to -0.09)∗	0.15
Ser	-1.19 (-1.74 to -0.63)∗∗∗	-0.23 (-0.78 to 0.33)	-1.09 (-1.64 to -0.53)∗∗∗	0.20
Glu	-3.70 (-6.02 to -1.37)∗∗∗	-0.28 (-2.61 to 2.04)	-0.61 (-2.94 to 1.71)	0.86
Pro	-1.56 (-3.42 to 0.30)	-0.86 (-2.72 to 1.00)	0.31 (-1.55 to 2.17)	0.69
Gly	-1.95 (-3.16 to -0.73)∗∗∗	-0.76 (-1.98 to 0.45)	0.10 (-1.11 to 1.31)	0.45
Ala	-1.62 (-2.87 to -0.36)∗∗	-0.22 (-1.47 to 1.03)	0.06 (-1.19 to 1.31)	0.46
Cys	-0.21 (-0.41 to -0.01)∗	−0.21 (-0.41 to -0.01)∗	-0.05 (-0.25 to 0.16)	0.07
Val	-1.90 (-2.94 to -0.85)∗∗∗	-0.35 (-1.39 to 0.70)	-0.34 (-1.39 to 0.71)	0.39
Met	-0.52 (-0.78 to -0.27)∗∗∗	-0.11 (-0.37 to 0.14)	-0.21 (-0.47 to 0.04)	0.10
Ile	-1.12 (-1.86 to -0.38)∗∗	0.08 (-0.66 to 0.82)	-0.16 (-0.91 to 0.58)	0.27
Leu	-2.51 (-4.01 to -1.01)∗∗∗	-0.41 (-1.91 to 1.09)	-0.97 (-2.47 to 0.53)	0.55
Tyr	-1.07 (-1.64 to -0.49)∗∗∗	-0.19 (-0.77 to 0.39)	-0.25 (-0.83 to 0.32)	0.21
Phe	-1.21 (-2.17 to -0.25)∗∗	-0.11 (-1.07 to 0.85)	-0.07 (-1.03 to 0.89)	0.35
His	-0.82 (-1.18 to -0.45)∗∗∗	-1.87 (-2.24 to -1.50)∗∗∗	-1.73 (-2.10 to -1.36)∗∗∗	0.14
Lys	-2.44 (-4.72 to -0.17)∗	0.82 (-1.45 to 3.10)	1.34 (-0.93 to 3.62)	0.84
Arg	-3.10 (-6.48 to 0.29)	1.36 (-2.03 to 4.74)	3.87 (0.49–7.26)∗	1.25

Negative sign in front of the mean indicates a decrease in the concentration of the amino acid in the treated sample compared to the control.

∗p ​< ​0.05, ∗∗p ​< ​0.01, ∗∗∗p ​< ​0.001.

#### 3.4.3 The effect of CDDP, PtNPs-10 and PtNPs-40 on the amino acid profile of liver and brain in chicken embryo

To determine the effects of CDDP, PtNPs-10 and PtNPs-40 on the amino acids profiles of liver and brain, we harvested the tissues after 24 ​h treatment and we analyzed their amino acids contents separately. In addition to the amino acids analysis of the CDDP and PtNPs treated and untreated metastasized tissues, we analyzed, the liver and brain of the samples which were not subjected to MDA-MB-231 ​cells xenograft (negative control), to determine whether the metastatic colonization induced changes in the amino acid contents.

The presence of a tumor on the surface of CAM did not induce any substantial changes in the amino acid profiles of the liver, except for the significant increase in the concentration of Thr. The 24 ​h treatments of the tumor bearing embryos did not result in any considerable changes besides the significantly lower values for Tyr upon the treatment with PtNPs-10 (Table 3). However, it is worth mentioning that CDDP and PtNPs-10 tend to reduce the amount of the amino acids, whereas in the case of PtNPs-40, such trend was not observed.

**Table 3:** Concentration of amino acids in the liver. Data is shown as mean difference of treated and untreated samples in a 95% (CI) and standard error (SE); n ​= ​9.

Amino acid	Mean difference (g/kg) at 95% CI				SE
	Untreated vs. NTC	CDDP vs. Untreated	PtNPs-10 vs. Untreated	PtNPs-40 vs. Untreated	
Asp	1.29 (-1.10 to 3.68)	-0.14 (-2.23 to 2.25)	-1.37 (-3.76 to 1.02)	0.63 (-1.76 to 3.02)	0.84
Thr	0.98 (0.04–1.93)∗	-0.83 (-1.77 to 0.11)	-0.10 (-1.04 to 0.84)	0.90 (-0.04 to 1.84)	0.33
Ser	0.37 (-0.66 to 1.40)	-0.38 (-1.41 to 0.66)	-0.35 (-1.38 to 0.69)	0.78 (-0.25 to 1.82)	0.36
Glu	1.40 (-1.46 to 4.26)	0.02 (-2.85 to 2.88)	-1.61 (-4.47 to 1.26)	0.83 (-2.03 to 3.69)	1.00
Pro	0.16 (-1.35 to 1.67)	-0.06 (-1.57 to 1.46)	-1.49 (-3.00 to 0.03)	-0.84 (-2.35 to 0.67)	0.53
Gly	0.64 (-0.62 to 1.90)	0.06 (-1.20 to 1.32)	-0.53 (-1.79 to 0.73)	0.26 (-1.00 to 1.52)	0.44
Ala	1.05 (-0.65 to 2.78)	-0.15 (-1.86 to 1.55)	-1.02 (-2.73 to 0.68)	0.19 (-1.52 to 1.89)	0.60
Cys	-0.05 (-0.17 to 0.08)	0.06 (-0.07 to 0.19)	-0.02 (-0.15 to 0.10)	0.03 (-0.10 to 0.16)	0.04
Val	1.15 (-0.63 to 2.93)	-0.32 (-2.10 to 1.46)	-1.11 (-2.89 to 0.67)	0.39 (-1.39 to 2.16)	0.62
Met	0.02 (-0.46 to 0.50)	0.27 (-0.22 to 0.75)	-0.33 (-0.81 to 0.16)	0.32 (-0.17 to 0.80)	0.17
Ile	0.49 (-0.62 to 1.61)	-0.05 (-1.16 to 1.06)	-0.60 (-1.71 to 0.51)	0.39 (-0.72 to 1.50)	0.39
Leu	1.23 (-0.87 (3.32)	-0.32 (-2.41 to 1.78)	-1.50 (-3.60 to 0.59)	0.26 (-1.84 to 2.35)	0.73
Tyr	0.29 (-0.45 to 1.03)	-0.13 (-0.87 to 0.61)	-0.79 (-1.53 to -0.05)∗	0.00 (-0.74 to 0.74)	0.26
Phe	0.85 (-0.38 to 2.08)	-0.37 (-1.60 to 0.86)	-1.15 (-2.38 to 0.08)	-0.04 (-1.27 to 1.19)	0.43
His	0.49 (-0.16 to 1.14)	-0.27 (-0.92 to 0.38)	-0.49 (-1.14 to 0.16)	-0.13 (-0.78 to 0.52)	0.23
Lys	1.56 (-0.69 to 3.81)	-0.48 (-2.73 to 1.77)	-1.52 (-3.77 to 0.72)	0.30 (-1.95 to 2.55)	0.79
Arg	0.90 (-2.06 to 3.86)	0.76 (-2.20 to 3.71)	0.6 (-2.20 to 3.72)	1.84 (-1.12 to 4.81)	1.04

Negative sign in front of the mean indicates a decrease in the concentration of the amino acid in the treated sample compared to the control.NTC- No Tumor Cells (Samples of chick embryo which were not xenografted with MDA-MB-231; negative control).

∗p ​< ​0.05, ∗∗p ​< ​0.01, ∗∗∗p ​< ​0.001.

Unlike the livers, which were not affected by the presence of a tumor, the brains displayed a completely different landscape, where the concentrations of most of the amino acids were significantly higher than the brains from the non-tumor bearing embryos (Table 4). Compared to these increased values of the untreated tumor bearing chicks, the treatment with CDDP induced a non-significant decrease of the amino acids. Leu was however the only amino acid which was significantly decreased. In the case of treatments with the PtNPs such amino acid depletions were not observed. In fact, the concentrations of most of the amino acids were elevated, with significant increases of Pro and Phe of the embryos treated with PtNPs-10, whereas the treatment with PtNPs-40 caused a significant increase of Phe and Arg.

**Table 4:** Concentration of amino acids in the brain. Data is shown as mean difference of treated and untreated samples in a 95% (CI) and standard error (SE); n ​= ​9.

Amino acid	Mean difference (g/kg) at 95% CI				SE
	Untreated vs. NTC	CDDP vs. Untreated	PtNPs-10 vs. Untreated	PtNPs-40 vs. Untreated	
Asp	0.94 (0.35–1.53)∗∗∗	-0.17 (-0.76 to 0.42)	0.31 (-0.28 to 0.90)	0.22 (-0.37 to 0.81)	0.21
Thr	0.35 (0.06–0.64)∗	-0.10 (-0.05 to 0.53)	0.04 (-0.25 to 0.33)	0.06 (-0.23 to 0.35)	0.10
Ser	0.54 (0.18–0.91)∗∗	-0.30 (-0.66 to 0.07)	-0.11 (-0.48 to 0.26)	-0.10 (-0.46 to 0.27)	0.13
Glu	1.19 (0.43–1.95)∗∗∗	-0.32 (-1.08 to 0.44)	0.34 (-0.42 to 1.10)	0.28 (-0.48 to 1.04)	0.27
Pro	0.20 (-0.64 to 1.04)	0.00 (-0.83 to 0.84)	1.42 (0.58–2.25)∗∗∗	0.79 (-0.25 to 1.63)	0.29
Gly	0.30 (0.06–0.53)∗∗	-0.16 (-0.40 to 0.07)	0.05 (0.18–0.29)	0.15 (-0.08 to 0.39)	0.08
Ala	0.50 (0.16–0.83)∗∗	-0.10 (-0.44 to 0.23)	0.11 (-0.23 to 0.44)	-0.01 (-0.35 to 0.32)	0.12
Cys	0.05 (-0.03 to 0.14)	-0.02 (-0.10 to 0.07)	0.07 (-0.01 to 0.16)	0.05 (-0.04 to 0.13)	0.03
Val	0.55 (0.21–0.88)∗∗∗	-0.21 (-0.54 to 0.13)	0.11 (-0.23 to 0.44)	0.15 (-0.18 to 0.49)	0.12
Met	0.39 (0.16–0.61)∗∗∗	-0.10 (-0.32 to 0.12)	0.05 (-0.17 to 0.27)	-0.12 (-0.35 to 0.10)	0.08
Ile	0.34 (-0.06 to 0.75)	-0.12 (-0.53 to 0.28)	0.10 (-0.31 to 0.50)	-0.05 (-0.46 to 0.35)	0.14
Leu	1.21 (0.86–1.57)∗∗∗	-0.53 (-0.88 to -0.17)∗∗	0.17 (-0.19 to 0.52)	0.33 (-0.03 to 0.68)	0.13
Tyr	0.31 (-0.14 to 0.76)	-0.20 (-0.65 to 0.25)	0.14 (-0.31 to 0.59)	0.18 (-0.27 to 0.63)	0.16
Phe	0.22 (-0.09 to 0.53)	-0.02 (-0.34 to 0.29)	0.35 (0.04–0.66)∗	0.35 (0.04–0.67)∗	0.11
His	0.29 (0.14–0.42)∗∗∗	-0.12 (-0.26 to 0.01)	0.07 (-0.07 to 0.2)	0.10 (-0.04 to 0.23)	0.05
Lys	0.82 (0.46–1.18)∗∗∗	-0.28 (-0.64 to 0.08)	0.14 (-0.22 to 0.50)	0.10 (-0.26 to 0.46)	0.13
Arg	-0.02 (-1.33 to 1.30)	0.31 (-1.00 to 1.63)	0.63 (-0.69 to 1.94)	1.81 (0.49–3.12)∗∗	0.46

Negative sign in front of the mean indicates a decrease in the concentration of the amino acid in the treated sample compared to the control.NTC- No Tumor Cells (Samples of chick embryo which were not xenografted with MDA-MB-231; negative control).

∗p ​< ​0.05, ∗∗p ​< ​0.01, ∗∗∗p ​< ​0.001.

### 3.5 Impact of CDDP, PtNPs-10 and PtNPs-40 on the expression of mRNA encoding the major enzymes of TCA cycle

**Fig. 5:**
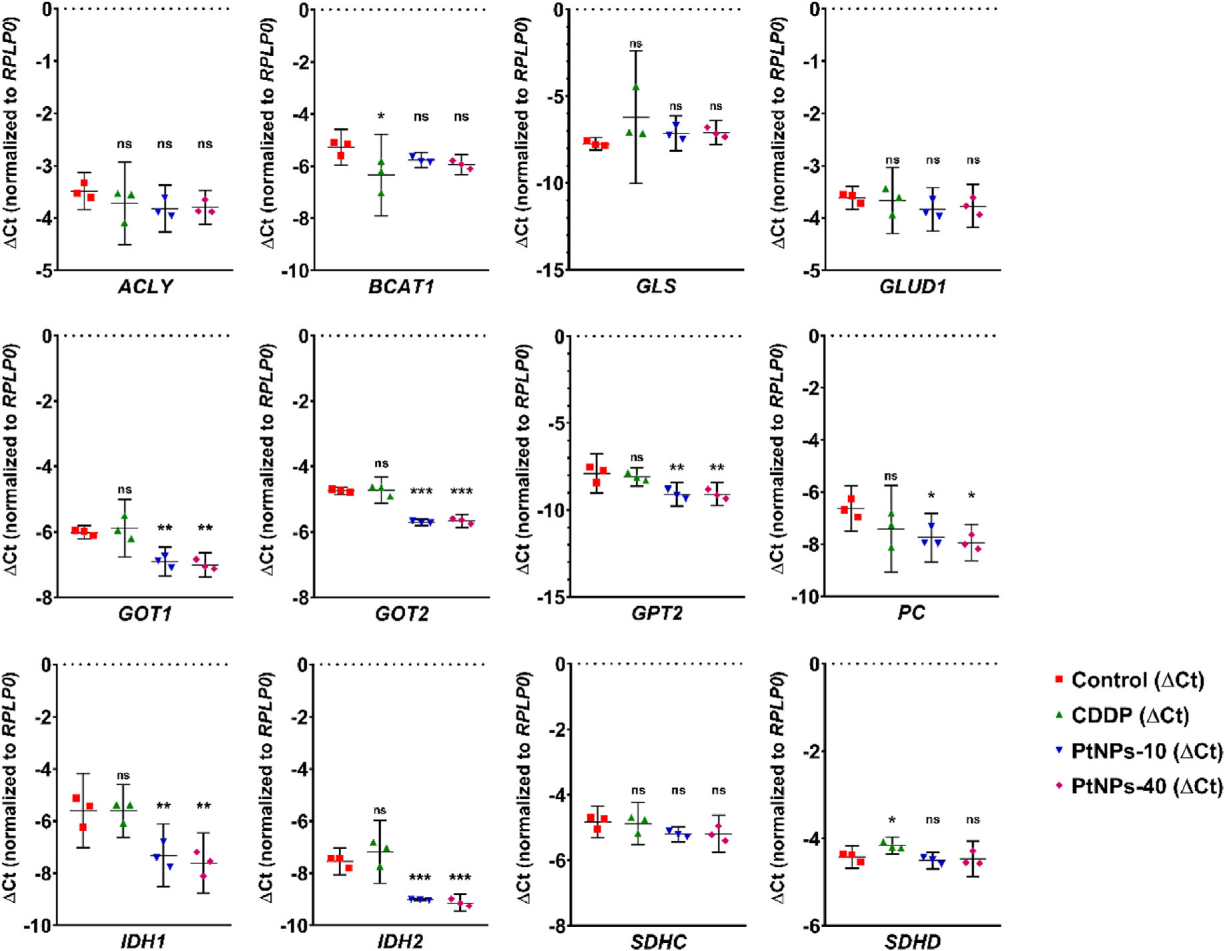
Relative gene expression for selected genes from TCA cycle in MDA-MB-231 ​cells treated with CDDP and PtNPs during 24 ​h compared with untreated cells. Data after normalization (ΔCT) are presented as a mean with 95% confidence interval from three independent biological replicates (n ​= ​3); ∗p ​< ​0.05, ∗∗p ​< ​0.01, ∗∗∗p ​< ​0.001.

Since the amino acids are directly related to the metabolic exchange in the TCA cycle, a major metabolic pathway that promotes tumor development, we examined the impact of the CDDP and PtNPs treatments to the main enzymes regulating the TCA cycle. In this context, MDA-MB-231 was treated with CDDP and PtNPs during 24 ​h, and subsequently we isolated RNA to analyze the expression of selected genes from TCA cycle by qRT-PCR. Significant down-regulation was observed in *GOT1, GOT2, GPT2, PC, IDH1*, and *IDH2* genes, upon the treatments with PtNPs-10 and PtNPs-40, with the most prominent decline in the expression of *GOT2* and *IDH2* genes (Fig. 5). Interestingly, the downregulation of these genes was consistent in both PtNPs treatments, but not in the samples treated with CDDP. On the other hand, a significant down-regulation of *BCAT1* and up-regulation of *SDHD* was noted in the CDDP treated samples, which was not observed upon the treatments with either of the PtNPs (Fig. 5).

## 4 Discussion

This study was designed to highlight the importance of CAM assay for the evaluation of the efficiency of CDDP, a conventionally applied chemotherapeutic compound, and PVP-PtNPs, as alternative treatments, as well to unravel their effect to the amino acid metabolism and pinpoint the unique targets of the related TCA cycle. The CAM assay proves efficient due to the lack of innervation and insufficient immunocompetency allowing a fast tumor growth [[27], [28], [29]]. The *ex ovo* installment provides an easily reachable large surface to observe the tumor's proportions as well as the embryo development [24]. However, the chances of survival are higher in the *in ovo* approach, and it provides the ability grow visible tumors to be extracted and evaluated by size and histological appearance [29,39]. In addition to the tumors, liver and brain were collected and subjected to further analyses [33,38].

The cytotoxicity of PtNPs has been examined repeatedly [[1], [2], [3],^7^,^30^], and previous studies have shown that it is based on intracellular processes such as mitochondrial and DNA damage upon endocytosis, resulting from lysosomal disintegration of the PtNPs into smaller fragments and release of Pt^+2^ [40]. Meanwhile, the presence of the polymer in the structure of the PtNPs imparts a high biocompatibility, minimizing the interaction with plasma protein which allows for a longer circulation and increased plasma-life of the PtNPs [[41], [42], [43]]. However, the molecular weight of the chosen polymer, has been shown to have implications into the magnitude of the toxicological effects, displaying higher toxicity of the PtNPs-10 which had smaller dimensions, compared to PtNPs-40 [30]. In fact, the molecular weight of the polymer is the main determinant of the rate of the disintegration of the nanoparticles, allowing faster release of the active drug when polymer with lower molecular weight is used, thus inducing higher toxicity [44]. Nevertheless, the antitumor efficiency of PtNPs *in vivo* has been rarely investigated, and mostly in murine models [8,10,45]. From our extensive research, it appears that data regarding CAM assay application for the investigation of PtNPs antitumor activity has been restricted to a sole study on glioblastoma multiforme [11]. Kutwin *et al.,* demonstrated a substantial weight and volume reduction of the tumors in both CDDP and PtNPs treated groups, as well as lower cell density of the tumor sections which was more noticeable in PtNPs treated tumors. Our PtNPs-10 and PtNPs-40 showed a superior potential to reduce tumor size compared to CDDP, and obvious disintegration zones with lower cell density were observed. Similar variations of the antitumor efficiency of CDDP against different tumors was revealed, where the comparison of primary tumors of several urothelial cancer cell lines showed substantial tumor reduction in some, but not in all the examined cell lines [46].

The cytostatic agents are known to decrease the extend of the overall vascular density, reducing the number of blood vessels that can reach the tumor, thus limiting the supply of nutrients [47]. Our study showed a correlation between the decreased vascular density and increased antitumor efficiency of the PtNPs-10 and PtNPs-40, and the less apparent effects of CDDP. The enhanced inhibitory effect on the tumor size by PtNPs may result from the unique ability of the nanoparticles to passively target cancer cells due to the enhanced permeability and retention (EPR) phenomenon, whereas molecules with low molecular weight are easily diffused back to the circulation and eliminated through the kidney [25,48,49]. Such improved effects of the nanostructured compositions have been confirmed even in different formulations of the same drug, i.e. CDDP, where nano-architectonic design of CDDP surpasses the effects of CDDP in its generic form [47,50].

The metastatic potential, the intravasation and extravasation in distant destination was determined through *ex ovo* and *in ovo* observation of CAM, as well, as fluorescent imaging of the chick embryo and liver and brain. Excellent visualization of the cells travelling through the blood vessels, as well as their colonization in both adjacent CAM and distal CAM from *ex ovo* and *in ovo* chick embryos, proved a greater inhibitory effect to the metastatic migration in PtNPs-10 treatment samples [29]. Wang *et al*.*,* described the mechanism of metastatic induction by PtNPs, as a disruption of the integrity of the endothelial membrane of the blood vessels, facilitating the intravasation and extravasation of the migrating breast cancer cells [51]. The fluorescent imaging of the primary tumors, as well as whole chick embryos agreed with these results, and together with the H & E section staining additionally provided indication of metastatic location in liver and brain [32]. Migrated cells were detected in both organs using a simplified method of a tissue pressed between two microscopic glass and subsequent imaging [33]. A novel methodology for thorough examination and assessment of *in vivo* cancer cell extravasation by directly injecting cancer cells into the CAM of chick embryos was proposed by Kim *et al*. [52]. The proposed *ex ovo* CAM assay is an efficient method which can be used to evaluate the potential antimetastatic effects of drugs that specifically target the cancer cell extravasation. However, using this method, the development of a 3D tumor on the CAM was unattainable, which was an essential requirement for the evaluation of the effects of the CDDP, PtNPs-10 and PtNPs-40 and their ability to suppress the growth of the tumor, intravasation from the primary tumor site, extravasation and metastatic spreading into other organs.

The application of various platinum-based drugs results in a disturbed amino acid metabolism, interference with the tRNA aminoacylation and protein synthesis [38,[53], [54], [55], [56]]. We analyzed the impact to the amino acid metabolism in MDA-MB-231 ​cells upon treatments with CDDP, PtNPs-10, and PtNPs-40 and we revealed a discernible contrast between the effects of CDDP and PtNPs. Specifically, an increase of the amino acids upon CDDP treatment was observed, whereas PtNPs showed decrease of the amino acids in significant proportions. These contrasting results imply a higher sensitivity of MDA-MB-231 ​cells towards PtNPs compared to CDDP, since an increase of the amino acids has been related to a degree of insensitivity to various chemotherapeutic agents [[57], [58], [59]], even when combinatorial effects of melittin or biguanide drugs with CDDP have been introduced [[60], [61], [62], [63]].

Regarding the amino acids’ profile in the primary tumors, CDDP resulted in a serious depletion of the whole amino acid set, which was completely inconsistent with the same treatment of the MDA-MB-231 ​cell culture. Such discrepancies could result from the fact that ideally precise excision of the tumors was difficult to attain, and the presence of CAM attached to the tumor could contribute to the contrast in the responses. On the other hand, the observation of less extensive, but similar trends of the impact to the amino acid profile between *in vitro* and *in vivo* experiments upon PtNPs treatments, suggests lower negative impact of the PtNPs to non-cancer tissues, probably arising from the EPR effect [25,48,49]. However, an interesting observation was the significant decrease of His, consistent upon all treatments, which could indicate a possible effect of CDDP and PtNPs on the angiogenetic regulation mediated through histidine-rich glycoprotein [64].

The less apparent alteration in the amino acid composition in liver and brain, may be related to the topical application of CDDP and PtNPs directly on the tumor, in combination with the ERP effect [25,48], and applying treatments for a longer duration could provide more significant results [38]. Despite this, we were still able to observe different trends in the responses of liver and brain. The trend of depletion of amino acids in liver upon CDDP and PtNPs-10 may point to an onset of toxicity, since most of the exogenously introduced substances are accumulated and metabolized in liver [65,66]. This will additionally reduce their delivery in other tissues, justifying the fact that the amino acids’ profile in brain seems to be unaffected by both PtNPs treatments, even though amino acid depletion in brain would be desired, due to the initial increase in tumor bearing embryos.

As extension to the alteration of the amino acid contents in the MDA cell culture, we analyzed the expression of the key enzymes involved in the TCA cycle of MDA-MB-231 ​cell line, and we established that the PtNPs have an extensive silencing effect on the genes of the TCA cycle, suggesting a substantial decline of the mitochondrial respiration [12,18]. Contrary to this, the minor effect of CDDP on the TCA cycle could explain the low impact on the amino acids profile of MDA-MB-231 ​cell culture. With respect to the downregulation of *GOT1*, *GOT2* and *GPT2* upon PtNPs treatments, it could impair the generation of α-ketoglutarate from Glu [17], which could impact the replenishment of Ala and Asp, and most importantly, the circulation of electron equivalents between the cytosol and mitochondria could be compromised. This would ultimately disrupt the redox balance of the MDA-MB-231 ​cells, affecting their ability to neutralize the generation of ROS [67]. The cumulative effect of the shortage of GOT1, GOT2 and GTP2 has been related to low survival of MDA-MB-231 ​cells, whereas their increased expression was considered a means to adapt for drug resistance [68,69]. Regarding the significant downregulation of *IDH1* and *IDH2*, an increased migratory ability of MDA-MB-231 ​cells has been associated with low levels of *IDH1/2* [15,70]. However, our embryos treated with PtNPs-10 and PtNPs-40, showed less metastasis than the control, as well as compared to CDDP. On the other hand, the overexpression of PC has been related to the increased metastatic potential of highly invasive MDA-MB-231 ​cell lines [71]. The embryos treated with PtNPs-10 and PtNPs-40 demonstrated a significant downregulation of *PC*, which may have been substantial to counteract the effects of *IDH1* and *IDH2*.

The treatment of the MDA-MB-231 ​cells with CDDP resulted in lower disruption of the TCA cycle, and interestingly, it affected different enzymes than PtNPs-10 and PtNPs-40, indicating a mechanism by different targeting. The SDHD subunit of the SDH complex displayed an increased expression in the CDDP treated cells. Upregulation of *SDHD* has been shown to be consistent with higher survival rates, which was induced by miRNA regulation of CDDP resistance [72,73] and high apoptotic effects have been associated with decreased SDH activity in various cell lines [74,75]. In contrast, high levels of *BCAT1* have been shown to promote the tumor growth and a decreased sensitivity to CDDP [[76], [77], [78]]. The tumors in our study were not significantly reduced, despite the decreased expression of *BCAT1* in MDA-MB-231 ​cells, suggesting that the reduced susceptibility to CDDP is reliant on the *SDHD* upregulation, which is consistent with the enhanced contents of the amino acids as well.

## 5 Conclusion

In this study, we evaluated the antitumor activity of CDDP and PtNPs in association with the impact on the amino acid metabolism in breast cancer by Chick Chorioallantoic Membrane (CAM) Assay. The combination of *in ovo* and *ex ovo* CAM assay provided an excellent platform which extended its applicability from studying carcinogenesis to the field of screening of anticancer activity of PtNPs and further study of the amino acids' fluctuations in liver and brain where metastatic expansions were detected. The PtNPs-10 has proven to be more successful in the reduction of the tumor growth than CDDP. We found out that the amino acid metabolism in MDA-MB-231 ​cell culture is the most susceptible to PtNPs-10. The evidence obtained from the observation of the effects on the amino acid's metabolism in the primary tumor, liver, and brain, highlights the higher adverse effects of CDDP to non-cancerous tissues. However, the PtNPs did not restore the amino acid profile that was altered by the established metastasis in the brain. In association with the alteration of the amino acid contents, the TCA cycle in MDA-MB-231 ​cells was greatly disrupted by both PtNPs-10 and PtNPs40, silencing a large set of enzymes that contributed to the higher cytostatic capacity of the PtNPs. The minor alterations of the expression of the TCA enzymes induced by CDDP, along with the maintained amino acid contents, point out to a lower sensitivity of MDA-MB-231 ​cells to this treatment. Therefore, we can assume that a different mechanism of action is employed by PtNPs, amplifying the sensitivity of the MDA-MB-231 to this treatment. Thus, we believe that the *in vivo* CAM model for characterization of PtNPs anticancer effect in breast cancer could contribute to the advance of personalized medicine practice in the future.

## Credit author statement

KM: Investigation, Validation, Formal analysis, Visualization, Writing - Original Draft; MR: Methodology, Investigation, Validation, Data curation, Writing – review & editing; NC: Methodology, Investigation, Validation; HM: Investigation, Validation, Data curation; ZS: Investigation, Validation, Formal analysis; DH: Investigation, Validation, Formal analysis; OZ: Writing – review & editing; ZH: Writing – review & editing; PK: Investigation, Validation, Visualization; VA: Resources, Writing – review & editing, Project administration, Funding acquisition; VM: Conceptualization, Supervision, Visualization, Writing - Review & Editing, Data curation. All authors contributed to the article and approved the submitted version.

## Declaration of competing interest

The authors declare that they have no known competing financial interests or personal relationships that could have appeared to influence the work reported in this paper.

## Data Availability

Data will be made available on request.
