# Supplementary material for: Chick chorioallantoic membrane (CAM) assay for the evaluation of the antitumor and antimetastatic activity of platinum-based drugs in association with the impact on the amino acid metabolism
Source: Mater Today Bio. 2023 Jan 31;19:100570. doi: 10.1016/j.mtbio.2023.100570 (PMC9941372; doi:10.1016/j.mtbio.2023.100570)
Supplement: Multimedia component 1 [file mmc1.docx]

Supplementary material

**Chick Chorioallantoic Membrane (CAM) Assay for the Evaluation of the Antitumor and Antimetastatic Activity of Platinum-based Drugs in Association with the Impact on the Amino Acid Metabolism**

Katerina Mitrevska^1^, Miguel Angel Merlos Rodrigo^1^, Natalia Cernei^1^, Hana Michalkova^1,2^, Zbynek Splichal^1,2^, David Hynek^1,2^, Ondrej Zitka^1,2^, Zbynek Heger^1,2^, Pavel Kopel^3^, Vojtech Adam^1,2^ and Vedran Milosavljevic^1,2*^

^1^ Department of Chemistry and Biochemistry, Mendel University in Brno, Zemedelska 1, CZ-613 00 Brno, Czech Republic, European Union.

^2^ Central European Institute of Technology, Brno University of Technology, Purkynova 123, CZ-612 00 Brno., Czech Republic, European Union.

^3^ Department of Inorganic Chemistry, Faculty of Science, Palacky University, 17. listopadu 12, CZ-779 00 Olomouc, Czech Republic

* Author to whom correspondence should be addressed; E-Mail: [vedran.milosavljevic@mendelu.cz](mailto:vedran.milosavljevic@mendelu.cz); Tel.: +420-545-133-249.


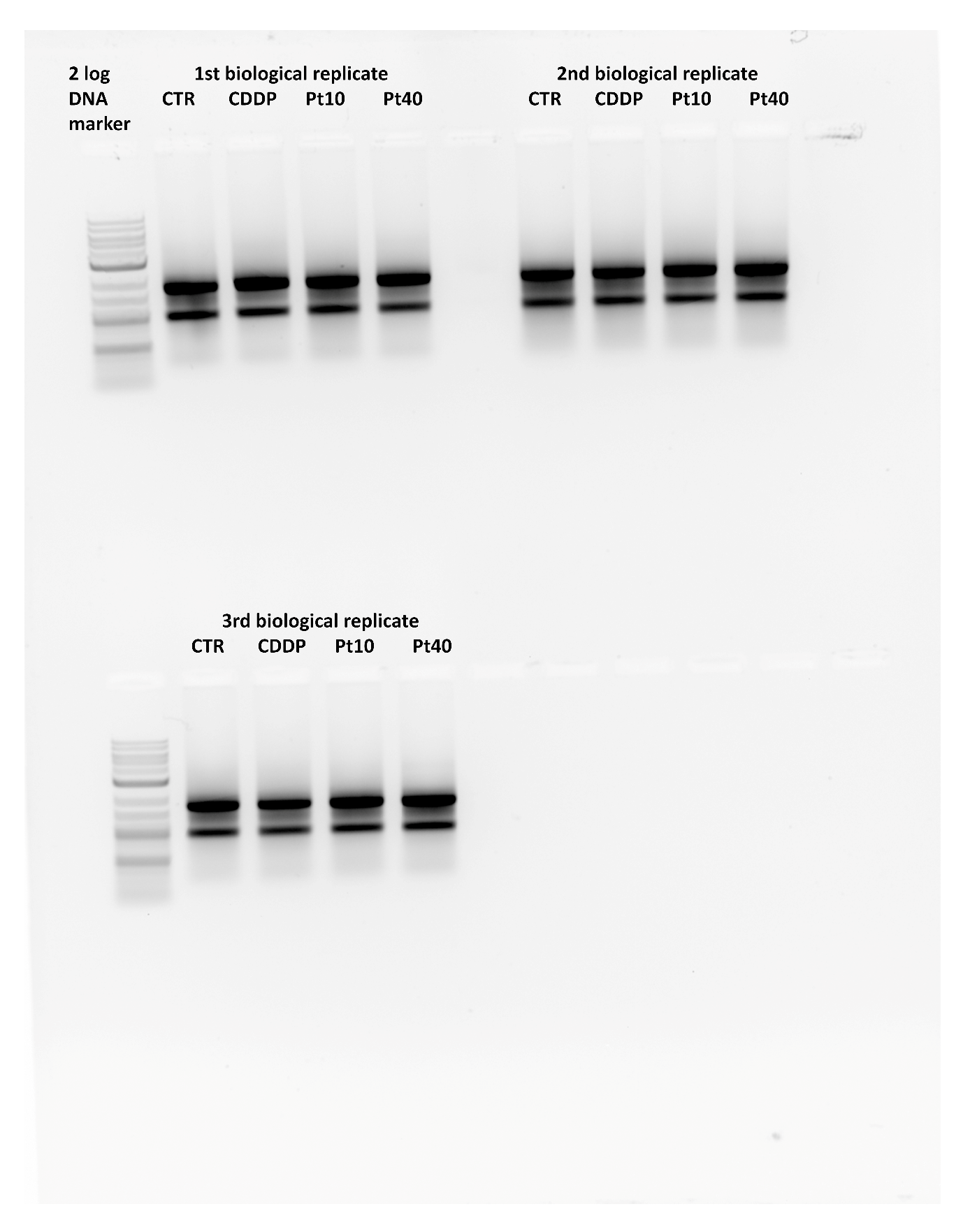


Figure S1. RNA integrity verified using a bleach gel

Table S1. List of qPCR Primers

| **GOI** | | | | | |
| --- | --- | --- | --- | --- | --- |
| **GENE SYMBOL** | **GENE NAME** | **PRIMER 5´-3´** | **NT** | **TYPE** | **AMPLICON SIZE (BP)** |
| **ACLY** | ATP citrate lyase | TGCACTGGAAGTAGAGAAGATTAC | 24 | flank intron | **117** |
|  |  | CGAGTAAAGGACCCACAGTTT | 21 |  |  |
| **BCAT1** | branched chain amino acid transaminase 1 | AAGCCCTGCTCTTTGTACTC | 20 | one exon | **111** |
|  |  | CACCTTTCCAGGCTCTTACAT | 21 |  |  |
| **GLS** | glutaminase | AGGTGGTGATCAAAGGGTAAAG | 22 | exon junction | **105** |
|  |  | TCCATGTCCATAGCTGACAAAG | 22 |  |  |
| **GLUD1** | glutamate dehydrogenase 1 | ATCGGGTGCATCTGAGAAAG | 20 | exon junction | **112** |
|  |  | CAGGTCCAATCCCAGGTTATAC | 22 |  |  |
| **GOT1** | glutamic-oxaloacetic transaminase 1 | CTGTCTATGTGTCCTCACCAAC | 22 | flank intron | **111** |
|  |  | CCAATCCTCTCTTCTCTGCATC | 22 |  |  |
| **GOT2** | glutamic-oxaloacetic transaminase 2 | GGCTTATATGGTGAGCGTGTAG | 22 | exon junction | **108** |
|  |  | GGAATACATGGGACGGATCAAG | 22 |  |  |
| **GPT2** | glutamic-pyruvic transaminase 2 | TCCTAAGGTGCTCTGCATAATC | 22 | exon junction | **136** |
|  |  | GTTGTCCTGGTACACCTCATC | 21 |  |  |
| **IDH1** | isocitrate dehydrogenase | GCTTGTGAGTGGATGGGTAAA | 21 | flank intron | **125** |
|  |  | CGTCACTTGGTGTGTAGGTTAT | 22 |  |  |
| **IDH2** | isocitrate dehydrogenase | GGAGATGGATGGTGATGAGATG | 22 | flank intron | **127** |
|  |  | ATCAGTCTGGTCACGGTTTG | 20 |  |  |
| **PC** | pyruvate carboxylase | CGACTCTGTGAAACTCGCTAAA | 22 | flank intron | **102** |
|  |  | GAGTTGACCTCGATGAAGTAGTG | 23 |  |  |
| **SDHC** | succinate dehydrogenase complex subunit C | CACTTGTCTTCCCTCTCATGTATC | 24 | flank intron | **121** |
|  |  | GTAAGAACCAGGACAACCACTC | 22 |  |  |
| **SDHD** | succinate dehydrogenase complex subunit D | CGAGAGGGTTGTCAGTGTTT | 20 | exon junction | **112** |
|  |  | CCAGTGACCATGAAGAGTGAG | 21 |  |  |
|  |  |  |  |  |  |
| **REFERENCE GENES** | | | | | |
| **GAPDH** | glyceraldehyde-3-phosphate dehydrogenase | CTTTGGTATCGTGGAAGGACTC | 22 | flank intron | **133** |
|  |  | AGTAGAGGCAGGGATGATGT | 20 |  |  |
| **PGK1** | phosphoglycerate kinase 1 | TCACTCGGGCTAAGCAGATT | 20 | flank intron | **201** |
|  |  | CAGTGCTCACATGGCTGACT | 20 |  |  |
| **RPLP0** | ribosomal protein lateral stalk subunit P0 | TCGACAATGGCAGCATCTAC | 20 | flank intron | **191** |
|  |  | ATCCGTCTCCACAGACAAGG | 20 |  |  |


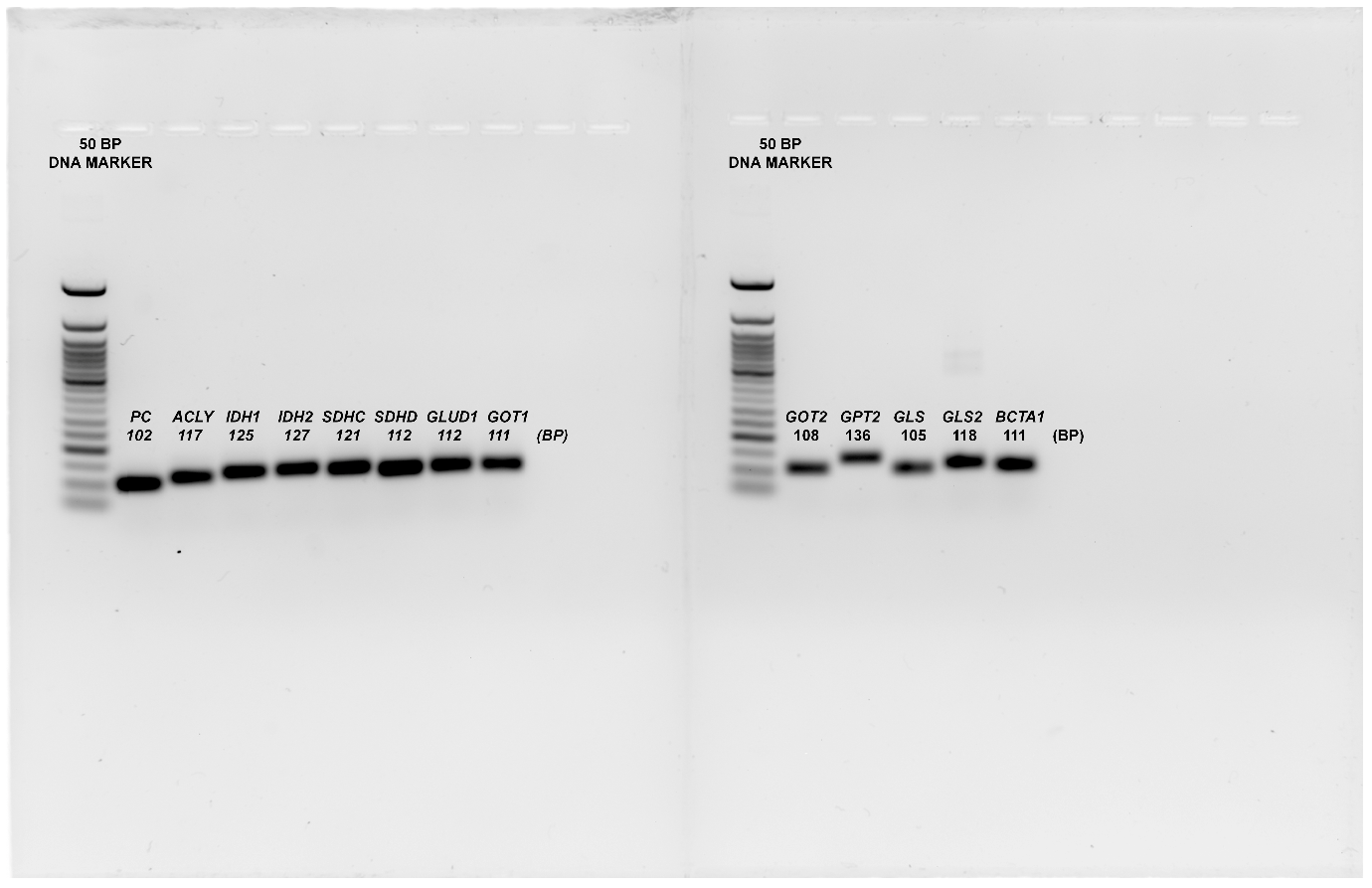


Figure S2. Validation of the amplicon size and control of primer-dimer formation.


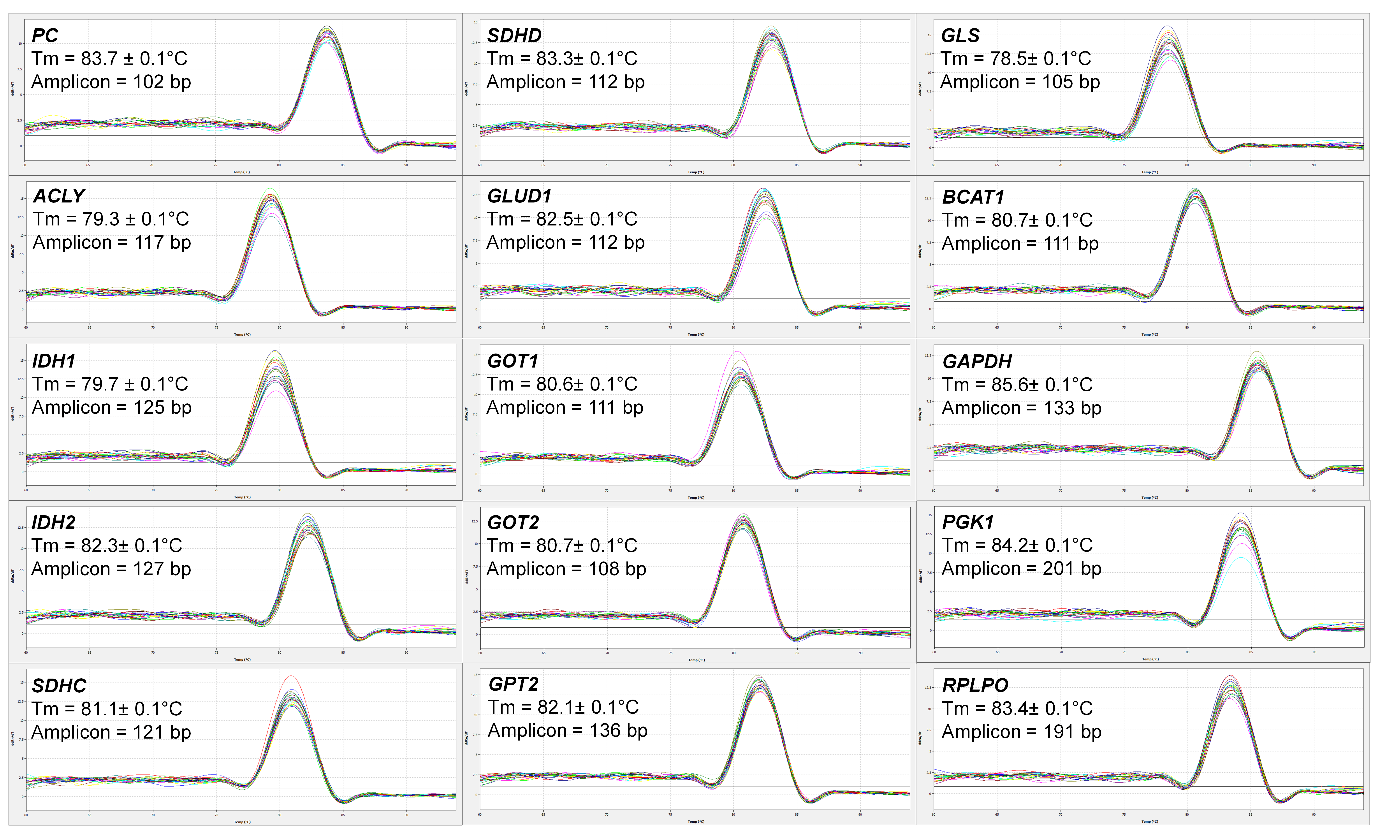


Figure S3. Mealting Curve Analysis after RT-qPCR: Tm of Amplicons (Mean±SD)

Table S2. CT data of housekeeping Genes by BestKeeper

|  | **RPLP0** | **GAPDH** | **PGK1** |
| --- | --- | --- | --- |
| **n** | **12** | **12** | **12** |
| **geo Mean [CT]** | **13.51** | **13.33** | **16.08** |
| **AR Mean [CT]** | **13.51** | **13.34** | **16.09** |
| **min [CT]** | **13.25** | **12.72** | **15.18** |
| **max [CT]** | **13.89** | **14.06** | **16.97** |
| **std dev [+/- CT]** | **0.15** | **0.25** | **0.25** |
| **CV [% CT]** | **1.14** | **1.87** | **1.53** |
| **min [x-fold]** | **-1.2** | **-1.53** | **-1.87** |
| **max [x-fold]** | **1.3** | **1.66** | **1.85** |
| **std dev [+/- x-fold]** | **1.11** | **1.19** | **1.19** |


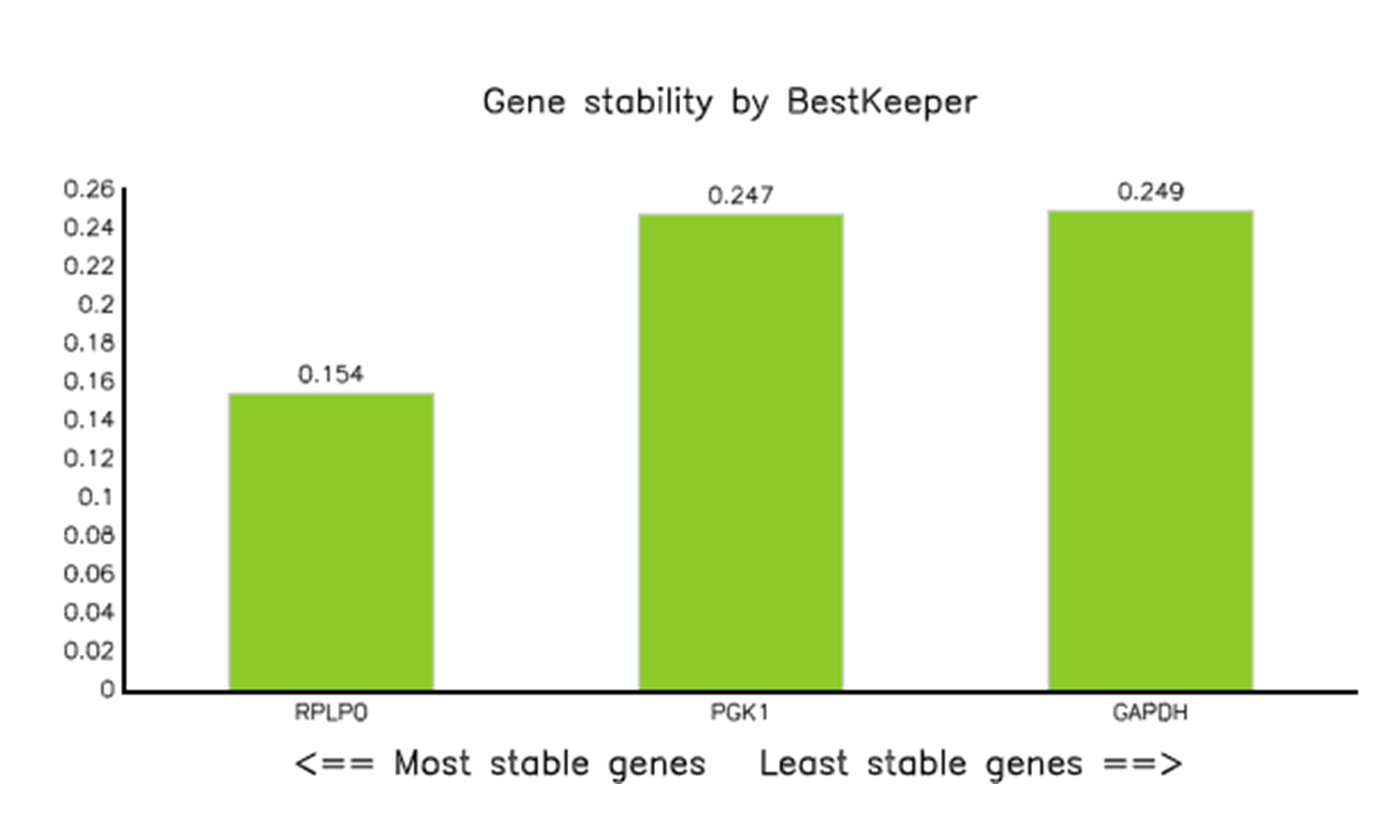


Figure S4. Stability of the selected reference genes by BestKeeper algorithm


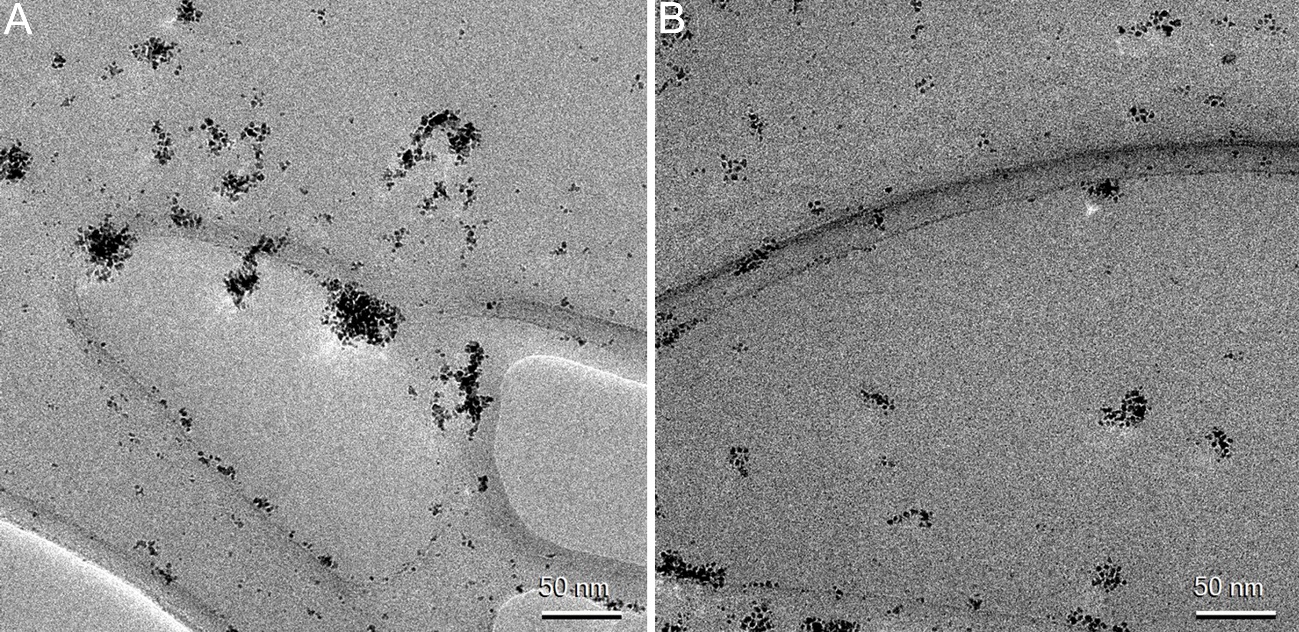


Figure S5. Characterization of PtNPs-10 (A) and PtNPs-40 (B) using TEM; scale bar 50 nm.


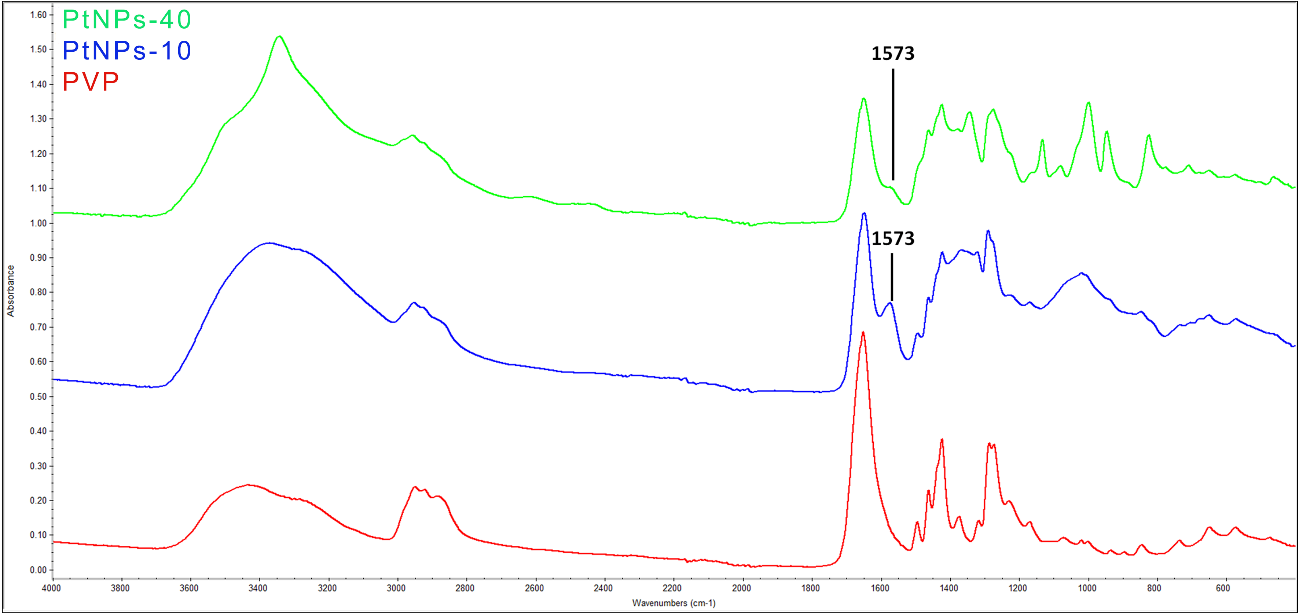


Figure S6. Characterization of PtNPs-10 and PtNPs-40 by recording the FTIR spectra


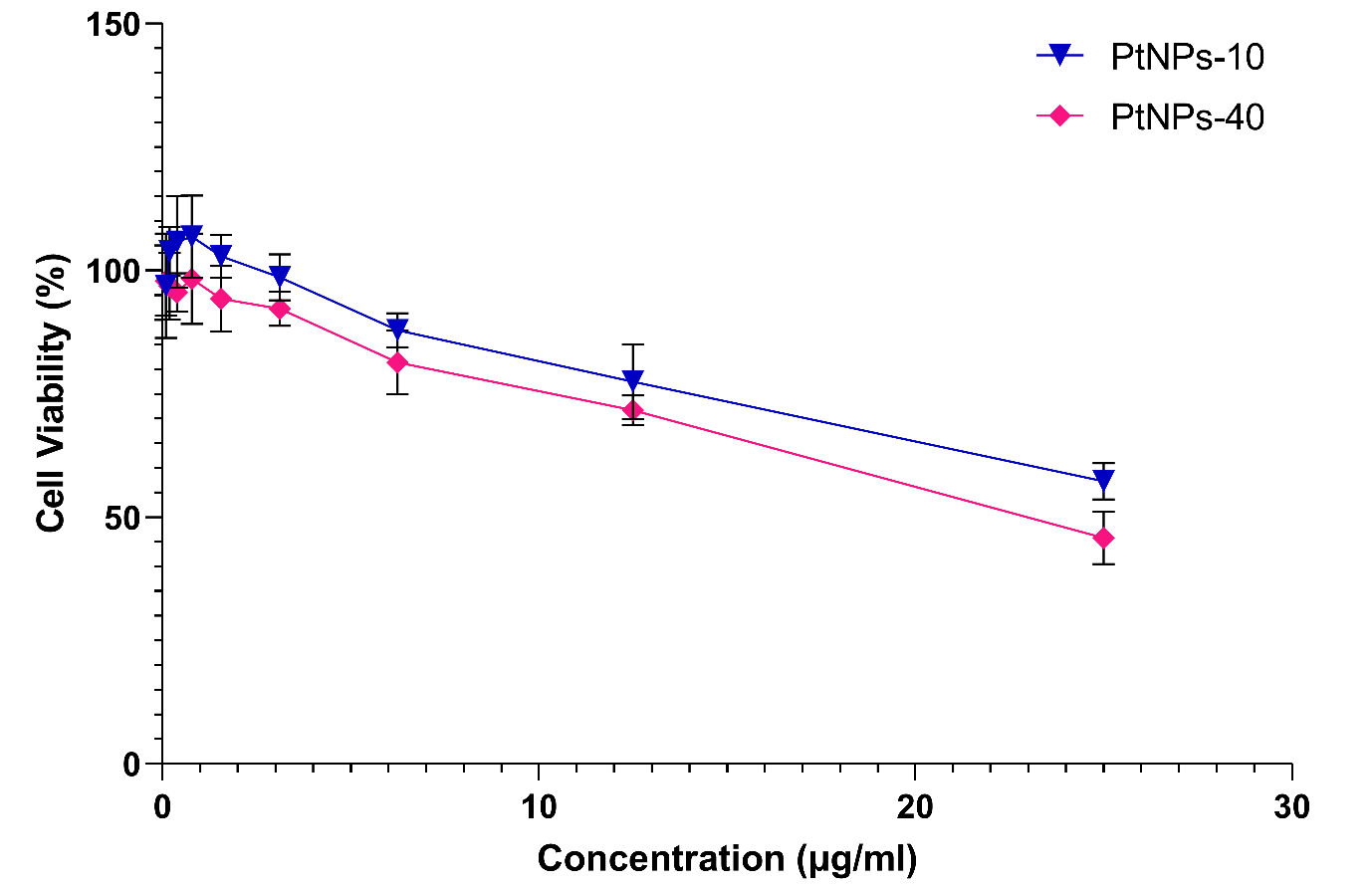


Figure S7. Concentration-dependent cell viability of MDA-MB-231 cell line against PtNPs-10 and PtNPs-40 obtained by MTT assay after 24 h treatment.


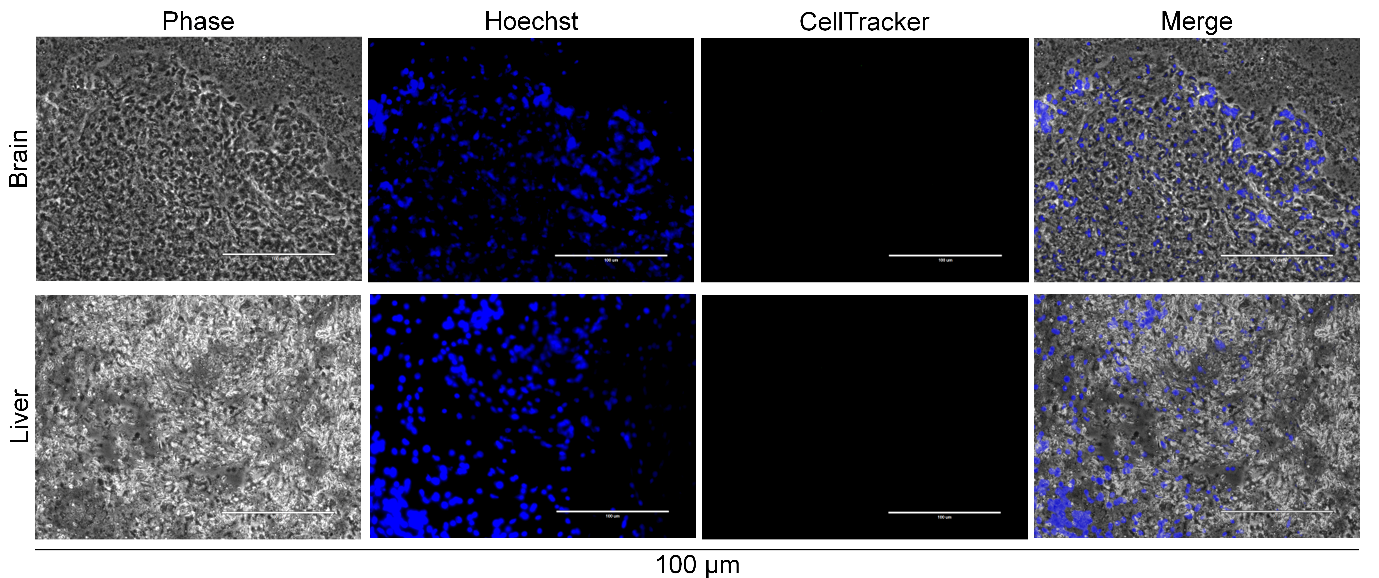


Figure S8. Fluorescent microscopic imaging of liver and brain without MDA-MB-231 xenographting (negative control), showing absence of cancer cells. Nuclei are labeled with Hoechst 33258 nuclei counterstain: scale bar 100 μm.
